# Amphipathic β‑Sheet-Forming Octapeptide Self-Assembly Using the Martini Potential Family

**DOI:** 10.1021/acsomega.6c02457

**Published:** 2026-08-12

**Authors:** Dibyajyoti Maity, Baofu Qiao

**Affiliations:** Department of Natural Sciences, Baruch College, City University of New York, New York, New York 10010, United States

## Abstract

Amphipathic peptides can self-assemble into β-sheet-rich fibrils and hydrogels. The potential applications of these nanomaterials in biomedicine, drug delivery, and tissue engineering have sparked significant research. Current computational screens for amphipathic peptides heavily rely on coarse-grained molecular dynamics (CGMD) simulations using the Martini force field to ascertain the self-assembly potential of novel peptide sequences. This approach, however, showed limited success in self-assembling amphipathic peptides into experimentally observed β-sheet-rich hydrogels. We have systematically investigated this phenomenon using FKFEFKFE, an amphipathic octapeptide with a solved high-resolution self-assembled three-dimensional (3D) structure, as our model system. We first performed all-atom molecular dynamics (AAMD) simulations, which support the essential role of neutral capping groups in reproducing the cryogenic electron microscopy (Cryo-EM) structure. Accordingly, CGMD simulations were conducted to compare the efficacy of the Martini versions 2.1, 2.2, 2.2P, and 3. Surprisingly, Martini 2.1 was the best at maintaining the self-assembled bilayer structure of the peptide observed in Cryo-EM. The self-assembly of 20 mM FKFEFKFE peptides from a random initial arrangement was then examined via 5 μs CGMD simulations, further confirming that Martini 2.1 can successfully simulate the formation of a β-sheet-rich bilayer with phenylalanine side chains embedded between the two layers. Moreover, modifications to the Martini 3 potential and the use of small water models, which were promising for shorter-peptide self-assembly, have limited success. We further showed that configurational entropy performs better at characterizing the assembly of ordered structures than the routinely used aggregation propensity score. Though the FKFEFKFE peptide was exclusively examined, the comprehensive benchmarking conducted here provides valuable insights into the factors influencing the CGMD of peptide self-assembly. Overall, the results support the development of robust, reproducible CGMD protocols and analysis tools for studying self-assembling peptides, enabling the discovery of novel supramolecular structures and biomaterials.

## Graphical abstract

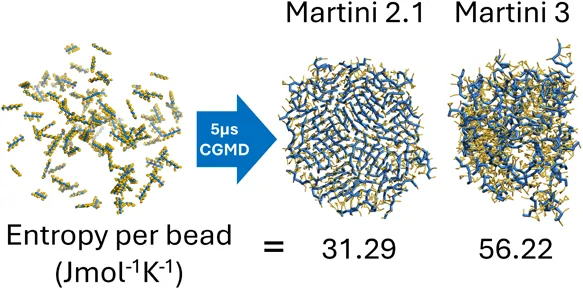

## Introduction

Self-assembling peptides spontaneously organize into well-defined, stable arrangements through noncovalent interactions.^1^ Due to their biocompatibility and biomimicry of physiological structures such as the extracellular matrix,^2–4^ self-assembling peptides and their hydrogels have numerous promising applications, especially in biomedicine, such as antimicrobials, tissue engineering, wound healing, vaccine adjuvant, and controlled delivery of anticancer and anti-inflammatory drugs.^5–10^ Self-assembling peptides have been the focus of significant research, and particular interest lies in amphipathic peptides with electrostatically self-complementary sequences that self-assemble into β-sheet-rich hydrogels.^11^ A prototypical example is the self-assembly of FKFEFKFE into a network of filaments comprised of left-handed helical ribbons of antiparallel β-sheets.^12,13^ Recently, this peptide has also been hybridized with another functional peptide to promote the healing of skin wounds.^14^ The N-acetylated and C-amidated FKFEFKFE peptide forms nanoribbons and nanotubes of varying pitch and width, comprised of twisted bilayers of inner parallel and outer antiparallel β-sheets.^15^ A fundamental understanding of the peptide sequence and self-assembly behavior is critical for the development of tunable peptide sequences with desired supramolecular structures.^16^

In this regard, the entire sequence space of 400 dipeptides^17^ and 8000 tripeptides^18^ has been screened for peptides with high aggregation propensity (AP) in water, using coarse-grained molecular dynamics (CGMD) simulations with the Martini 2.1 and 2.2 force fields, respectively. AP score is the ratio of the solvent accessible surface area (SASA) before and after the peptide assembly through molecular dynamics (MD) simulations, starting with a random arrangement of peptides in the simulation box. An AP score greater than 1 indicates aggregation, since the SASA decreases when multiple peptides come together, expelling solvent. It was quickly realized that this brute-force approach is impractical for longer peptides, as the search space grows exponentially, and the focus has shifted toward the development of AI models to search for novel self-assembling peptides. Limited availability of experimentally labeled data sets, with a lack of harmonization, significant positive class imbalance, and the strong dependence of the self-assembled morphology on experimental conditions such as solvent, concentration, pH, temperature, and functional groups protecting the peptide termini, make this extremely challenging.^19^ Still, active learning, human-in-the-loop machine learning (ML), recurrent neural networks, and transformers have shown considerable success in the search for self-assembling peptides with a sequence of length up to 24 amino acids using training data of AP scores generated from CGMD.^20–25^ Even with the advent of AI, MD remains relevant for validating predicted peptides and for simulating the self-assembly process to understand kinetics and energetics under various conditions.

All-atom molecular dynamics (AAMD), although more accurate, is unable to reach the time scales required to observe the self-assembly of peptides from a random configuration.^26^ Instead, CGMD has tremendous potential for computationally studying supramolecular peptide assembly because of the 2–3 orders of magnitude speed-up from the simpler representation of molecules.^26,27^ The Martini CG potential, initially developed to simulate lipids and surfactants, has become the most widely used coarse-grained force field for CGMD.^28,29^ The Martini potential has also achieved great success in peptide self-assembly and has shown good agreement with microscopy-observed nanostructures.^26^ However, different versions of Martini show drastically varied morphology of the self-assembled FF dipeptide, with Martini 2.1 having the highest AP score.^30^ Similarly, Martini 2.1 was successful in reproducing the coassembly of FF dipeptide and FFF tripeptide in aqueous environments, whereas Martini 2.2 failed.^31^ A similar assessment on longer peptides remains unexplored. Given the importance of the Martini force fields in CGMD of peptide self-assembly and data generation for the development of AI tools for peptide predictions, we find it prudent to critically evaluate the Martini force field family for reliable simulation and reproduction of self-assembled peptide structures, especially for longer β-sheet-forming peptides.

In this study, we present a systematic evaluation of the stability and reproducibility of peptide self-assembly using the Martini force fields 2.1,^29^ 2.2,^32^ 2.2P (polarizable version),^33^ and 3 with FKFEFKFE as the model system.^34^ To the best of our knowledge, this is the only octapeptide with an electrostatically self-complementary sequence whose experimentally solved self-assembled structure is available in the Protein Data Bank (PDB) with the ID 7LQF. First, we evaluated the effect of peptide termini on the stability of the self-assembled structure from 1 μs AAMD with three different peptide termini: protected neutral termini, unprotected neutral termini, and unprotected charged termini. The AAMD showed that peptides with neutral termini maintained their self-assembled structure throughout the trajectory, demonstrating the importance of the capping groups and pH on peptide self-assembly.

Second, we performed CGMD simulations of the systems with unprotected neutral termini for 5 μs using Martini 2.1 and 2.2, and for 1 μs using Martini 2.2P and 3. In addition, we employed two different protocols with each Martini version, one with the traditionally recommended Berendsen barostat for Martini 2 simulations, and the other with the Parinello–Rahman barostat. The results indicated that Martini 2.1 with the Berendsen barostat outperformed the other combinations in maintaining the bilayer structure of FKFEFKFE observed in cryogenic electron microscopy (Cryo-EM). Third, we performed 5 μs CGMD to simulate the self-assembly process of 100 FKFEFKFE peptides starting from a random arrangement with a concentration of around 20 mM, which confirmed that Martini 2.1 can successfully simulate the self-assembly into a β-sheet-rich bilayer with the phenylalanine side chains positioned in the interior. Finally, we determined if the inability of Martini 3 to form self-assembled structures could be alleviated by modifying the phenylalanine side chain beads and using small water beads, as recommended for FF dipeptide self-assembly.^27,30^ Unlike previous studies, which focused solely on peptide aggregation and maximizing AP score, we focus on detecting peptide self-assembly in CGMD using configurational entropy, which is capable of discriminating between amorphous aggregation and self-assembly of ordered structures.

## Methods

### All-Atom Self-Assembled Structure Preparation

The Cryo-EM structure of the FKFEFKFE peptide (PDB ID: 7LQF) was downloaded. The structure is composed of four β-sheets, each pair forming a bilayer, and the two bilayers are adjacent to each other (Figure 1A). Three peptides from the ends of each β-sheet were removed to reduce the system size, leaving 10 peptides per β-sheet for a total of 40. In the PDB structure, the peptides are protected with acetyl and amide groups at the N- and C-termini, respectively, and the *N*-acetyl phenylalanine is assigned the 3-letter code 5CR, and the C-amide glutamate is named GMA. The atoms of the *N*-acetyl group were renamed as follows: CAA → CH3, CAL → C, and OAB → O, and were assigned ACE as the residue name. The remaining 5CR atoms corresponding to the phenylalanine residue were renamed PHE. Similarly, GMA was renamed to GLU, and the atoms with nonstandard names were fixed as follows: O → OE1, OXT → OE2, O1 → O, C → CD, and CD → C. The C-terminal amide nitrogen with the atom name N2 was removed to obtain the structure of the *N*-acetyl peptide (Ac-FKFEFKFE).

**Figure 1:**
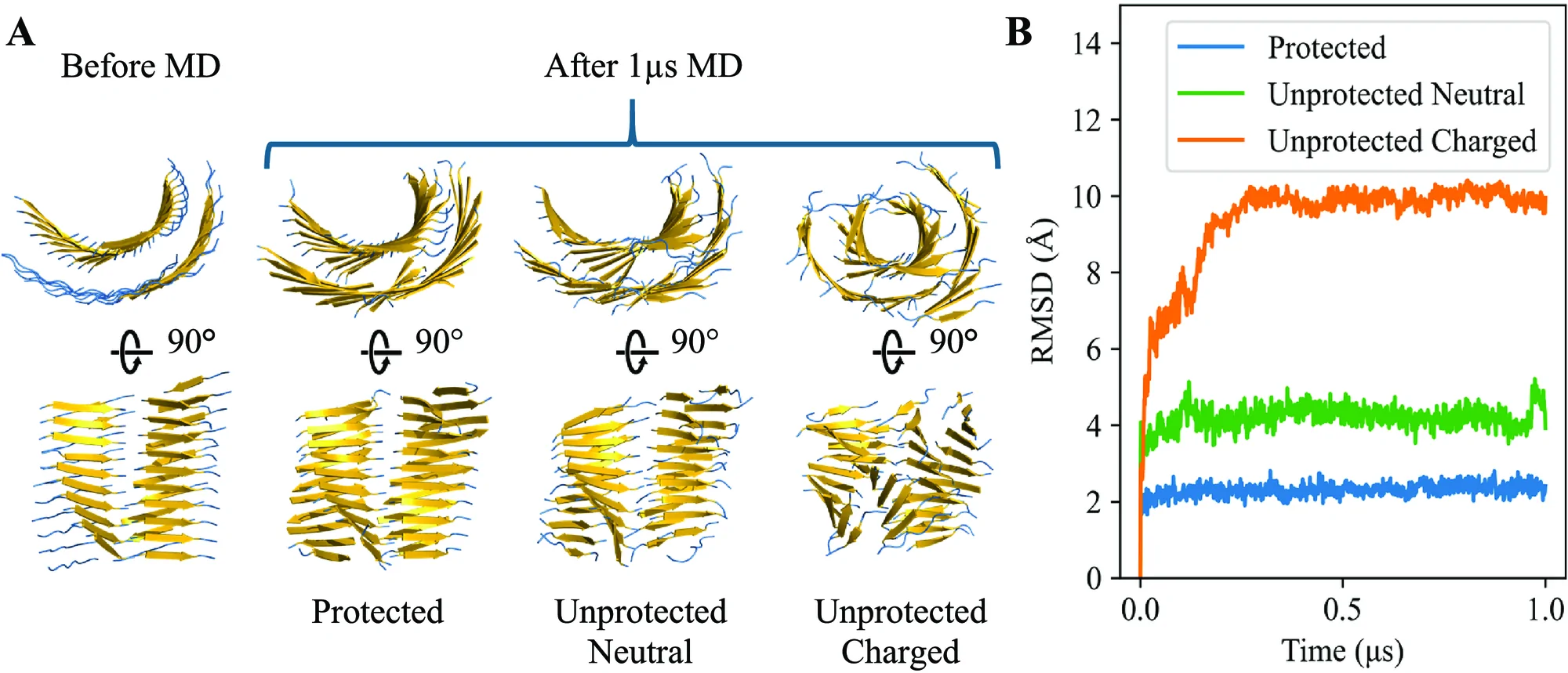
Charge-neutral termini preserved the left-handed helical bilayer structure of FKFEFKFE in the AAMD. (A) The starting structure (PDB ID: 7LQF) and frames at the end of 1 μs simulations with different termini. The structure has been colored according to the secondary structure, with yellow for β strands and blue for loops. The neutral (protected or unprotected) peptides remained stable throughout the simulation. In contrast, the charged peptides twisted remarkably, adopting a smaller-sized structure. (B) Root-mean-square deviation (RMSD) of the bilayers indicates that all the structures reached the equilibrium structure within 0.3 μs, and peptides with neutral termini greatly preserved the initial configuration.

### AAMD of the FKFEFKFE Self-Assembled Structure

The AAMD simulations of the three systems: Ac-FKFEFKFE-NH_2_ (*N*-acetyl C-amide FKFEFKFE), FKFEFKFE with neutral termini, and FKFEFKFE with charged termini (Figure S1) were performed using GROMACS 2024.4 and the CHARMM 36m force field^35^ to ascertain the stability of the self-assembled structure.^36^ An earlier version of the CHARMM potential (param19) was employed in AAMD of the FKFEFKFE peptide.^37^ CHARMM 36m is the latest version and was improved for the study of peptides and intrinsically disordered proteins.^38^ The primary advantage of starting from a self-assembled structure is that AAMD can explore the local conformational landscape of the system, whereas attaining this structure from a random arrangement of peptides is impossible in a reasonable time frame. The amidated C-terminus has already been defined as CT2. Thus, the Ac-FKFEFKFE-NH_2_ system was prepared from Ac-FKFEFKFE using the GROMACS command *gmx pdb2gmx* with interactive terminal selection (-ter option) and selecting CT2 for the C-termini of the peptides. Similarly, FKFEFKFE with neutral termini was prepared by selecting NH_2_ as the N-termini and COOH as the C-termini. FKFEFKFE with charged termini was prepared with the default capping groups of NH_3_^+^ and COO^–^ for the N- and C-termini, respectively.

Each system was placed in the center of a cubic box with an edge length of 9.53 nm, ensuring at least 1 nm between the peptide structure and the box edges, solvated with CHARMM TIP3P water, and subjected to steepest-descent energy minimization until all forces in the system were less than 1000 kJ/mol/nm. Since the peptides are charge-neutral, counterions were not required to neutralize the system, and no extra salt was added. Next, 100 ps NVT equilibration was performed at 300 K with temperature coupling to a velocity rescaling (V-rescale) thermostat with a time constant of 1 ps, followed by 100 ps NPT equilibration using the stochastic cell rescaling (C-rescale) barostat. In the production simulation, the neighbor searching was calculated up to a cutoff distance of 12 Å via the Verlet particle-based algorithm and was updated every 10th time step. The short-range electrostatic interactions were truncated at a distance of 12 Å, with the long-range interactions calculated using the particle mesh Ewald algorithm.^39,40^ The Lennard-Jones 12-6 interactions were switched off from 10 to 12 Å via the force-switch method. The temperature was managed using the V-rescale algorithm with the temperature of the system coupled at 300 K with a characteristic time of 1 ps. The pressure coupling was employed using the C-rescale algorithm at the reference pressure of 1 bar with a compressibility of 4.5 × 10^–5^ bar^–1^ and a constant of 5.0 ps. The integration time step of 2 fs was used with all the hydrogen-involved covalent bonds constrained using the LINCS algorithm.^41,42^ These parameters were recommended for the accurate reproduction of the original CHARMM simulation on lipid membranes^43^ and have been verified in our simulations on proteins,^44–46^ peptides,^47,48^ and peptide-based polymers^49–51^ that are of relevance to this work. The simulations were saved every 0.1 ns for data analysis. Each production simulation lasted for a duration of 1 μs. The root-mean-square deviation (RMSD) was calculated from the MD trajectory with respect to the first frame, and the final structures are provided in Figure 1A.

### CGMD of FKFEFKFE Self-Assembled Structure

The CG models of the systems for simulation with Martini 2.2, 2.2P, and 3 were generated from the all-atom structure of FKFEFKFE with neutral termini using the *Martinize2* program.^52^ *Martinize2* requires the secondary structure of protein/peptides to be specified; random coil was selected as the secondary structure with the “-ss C” option. Despite experimentally knowing that the peptides adopt β-strand secondary structure, we refrained from choosing β-strand (option of -ss E), which has been reported to be able to bias the self-assembly of peptides.^53^ In addition, the neutral termini and no side chain corrections were specified using the “-nt” and “-noscfix” options, respectively. *Martinize2* does not have an option to specify the Martini 2.1 force field; hence, the Martini 2.1 model was obtained from the Martini 2.2 model by changing the phenylalanine side chain beads from SC5 to SC4 in the topology file generated by *Martinize2*, which is the only difference between the particle type definitions in the two force fields for the amino acids present in the system. Furthermore, this approach utilizes 1250 kJ/mol/nm^2^ and 20 kJ/mol/rad^2^ as the force constants for backbone bonds and angles, respectively, as opposed to 400 kJ/mol/nm^2^ and 25 kJ/mol/rad^2^ introduced in the Martini 2.1 peptide topology generated by the *martinize.py* script. We found that the different force constants lead to negligible impacts on the obtained structures. Martini 2.3P is not investigated here because the only change between Martini 2.2P and 2.3P is the cation–π interaction with choline in phospholipids, which is absent in this work.

The Martini simulations with the Berendsen barostat were conducted according to the protocol and GROMACS MD parameters (.mdp files) in the High-throughput peptide self-assembly Martini 2 tutorial (https://cgmartini.nl/docs/tutorials/Legacy/martini2/high_throughput.html). Each system was placed in the center of a cubic box with an edge length of 17.26 nm, ensuring at least 5 nm between the peptide structure and the box edges, solvated with CG water, and energy-minimized for 5000 steps—finally, 1 μs of CGMD at 300 K with a V-rescale thermostat and a Berendsen barostat. In the Martini 2.1 and 2.2 simulations, 10% of the water beads were replaced with antifreeze water beads to prevent freezing; polarizable water was used in the Martini 2.2P simulation. The time for all CGMD results is given as time step × number of steps; the “effective time” would be 4 times as much, using the standard conversion factor accounting for the speed-up of the diffusion dynamics of water in CGMD.^28^

Given that the Berendsen barostat was employed when developing the early Martini versions (2.1,^29^ 2.2,^32^ and 2.2P^33^) for amino acids, and the Parrinello–Rahman barostat was used for the more recent versions (2.3P^54^ and 3 ^34^), we also examined the impact of the barostat here. Parrinello–Rahman barostat simulations were conducted following the Martini 3 tutorial for proteins (https://cgmartini.nl/docs/tutorials/Legacy/martini3/ProteinsI_Jan2025/). A short energy minimization of 100 steps was performed in vacuum, followed by solvation with water and another 100-step energy minimization. Next, the systems were equilibrated at 300 K for 200 ps using the Berendsen thermostat and barostat. Finally, a 1 μs CGMD at 300 K with a V-rescale thermostat and a Parinello–Rahman barostat was performed. A Martini 2.1 CGMD was also conducted using the C-rescale barostat following the Parinello–Rahman protocol described above. The Martini 2.1 and 2.2 simulations were extended to 5 μs when the systems appeared stable at the end of 1 μs CGMD.

The RMSD of the peptides relative to the starting structure was calculated to monitor deformation, and the final frames were visualized using MartiniGlass^55^ and VMD.^56^ The SASA of a single linear FKFEFKFE CG peptide in Martini 2 and 3 is 13.988 and 13.839 nm^2^, respectively. The AP score was calculated by dividing the SASA of 40 individual peptides, *i.e*., 559.52 nm^2^ (40 × 13.988 nm^2^) for Martini 2 and 553.56 nm^2^ (40 × 13.839 nm^2^) for Martini 3, by the SASA at a particular time. The configurational entropy of the backbone beads was calculated from the eigenvectors of the covariance matrix obtained using the GROMACS tool *gmx covar* and analyzed using *gmx anaeig* from the quasi-harmonic approach.^57^ The reported configurational entropy data have been normalized using the number of backbone beads for the convenience of comparison. The radial distribution function (RDF) of the backbone beads excluding the intramolecular peaks was calculated with *gmx rdf* from the last 500 ns of the trajectories.

### CGMD of 100 FKFEFKFE Self-Assembly Process

An all-atom linear FKFEFKFE peptide structure was created using the “Build Structure” menu in UCSF ChimeraX.^58^ The “add peptide” option was selected, and the amino acid sequence was provided. The backbone dihedral angles φ and ψ were set to −139 and 135°, respectively, corresponding to antiparallel β-sheets in the Dunbrack rotamer library.^59^ Accordingly, the CG model of the peptide was obtained using *Martinize2*, and 100 CG peptides were placed randomly in a cubic box with an edge length of 20 nm using the GROMACS command *gmx insert-molecules*. This system was simulated using Martini 2.1, 2.2, and 3. In each case, the system was energy minimized, equilibrated in the NVT ensemble for 1 ns at 300 K with a V-rescale thermostat, followed by NPT equilibration for 1 ns with a Berendsen barostat and V-rescale temperature coupling. Finally, a CGMD of 5 μs was performed using the Berendsen barostat.

Three additional CGMD in Martini 3, 5 μs each, was performed (1) using small water beads by changing the bead type in the topology from W to SW, or (2) changing the two TC5 beads in the phenylalanine side chain to SC4 (replicating the phenylalanine side chain in Martini 2.1) in the peptide topology file, or (3) combining the two, *i.e*., phenylalanine with SC4 beads in small water SW. All other steps remained unchanged in these simulations. The RMSD, RDF of backbone, SASA, AP score, and configurational entropy were calculated as described above to assess peptide self-assembly quantitatively. The configurational entropy was also evaluated using growing time windows to determine its convergence (Figure S2). The trajectory snapshots at 100 ns, 200 ns, 1 μs, and 5 μs are provided in Figures S3–S6 to monitor the evolution of the self-assembly process.

### CGMD of FKFEFKFE Nanotube

The structure of the FKFEFKFE nanotube with the PDB ID 7LQG was downloaded. The N-capped acetyl group was removed using PyMOL, and the structure was converted to a CG model with *martinize2* following the procedure described in the section on CGMD of FKFEFKFE Self-Assembled Structure above. The simulation parameters and protocol were the same as the CGMD of the 100 FKFEFKFE self-assembly process in Martini 2.1 with the Berendsen barostat as described above.

## Results

### Neutral Termini are Necessary for the Stability of the Self-Assembled Structure

All three 1 μs AAMD of the FKFEFKFE self-assembled bilayer structure with protected neutral termini (Ac-FKFEFKFE-NH_2_), unprotected neutral termini, and unprotected charged termini converged and attained a stable structure. The final frames from the AAMD of Ac-FKFEFKFE-NH_2_ and FKFEFKFE with neutral termini show that the initial curved bilayer of the nanoribbon Cryo-EM structure is preserved at the end of 1 μs AAMD with minimal deformation. In contrast, the curvature of the bilayer increased for FKFEFKFE with charged termini (Figure 1A). RMSD also indicates that both Ac-FKFEFKFE-NH_2_ and FKFEFKFE with neutral termini converged within a few nanoseconds and maintained the conformation throughout the 1 μs AAMD. In contrast, FKFEFKFE with unprotected charged termini enhanced the curvature and converged within 0.3 μs (Figure 1B). Thus, neutral termini are essential for favorable interactions between the two adjacent β-sheet bilayers and ensuring the stability of the systems. Hence, pH close to the isoelectric point of the peptides promotes the widening of the nanoribbons as well as larger diameter nanoribbons in agreement with the around 8 and 10 nm diameter of Ac-FKFEFKFE-NH_2_ nanoribbons at pH 4 and 7, respectively.^60^

### Martini 2.1 is Best at Preserving the FKFEFKFE Self-Assembled Structure

Inspired by the AAMD simulations, the unprotected neutral-termini peptide was exclusively examined in the subsequent CGMD simulation. Martini 2.1 maintained the β-sheet bilayer throughout the 5 μs simulations, irrespective of the barostat, with Berendsen and Parrinello–Rahman performing better than C-rescale, as evident from the highest AP score of about 2.7 and lowest entropy per backbone bead of around 23 J/mol/K (Figure 2). The peptides at the ends of the bilayer showed the greatest mobility, with some migrating while still attached to the edges of the bilayer. They also flipped from the top leaflet to the bottom one and *vice versa*. The elevated mobility of the edge peptides is expected to be suppressed by the closed tubular structure^15^ in the practical conditions. All three Martini 2.1 simulations, Martini 2.2 with Parinello–Rahman barostat, and Martini 2.2P with Berendsen barostat maintained a SASA close to or below that of the starting structure. Whereas the other simulations exhibited a higher SASA in agreement with the observed disorder and deformation compared to the starting structure (Figure S7). The ordered structure in Martini 2.1 and 2.2 is also reflected in the backbone RDF (Figure S8). Interestingly, the backbone RDF of Martini 2.2 and Martini 2.2P are significantly different despite observed similarities in the final structure. As opposed to the remarkable impacts of the CG potential, the barostat only marginally influenced the backbone RDF.

**Figure 2:**
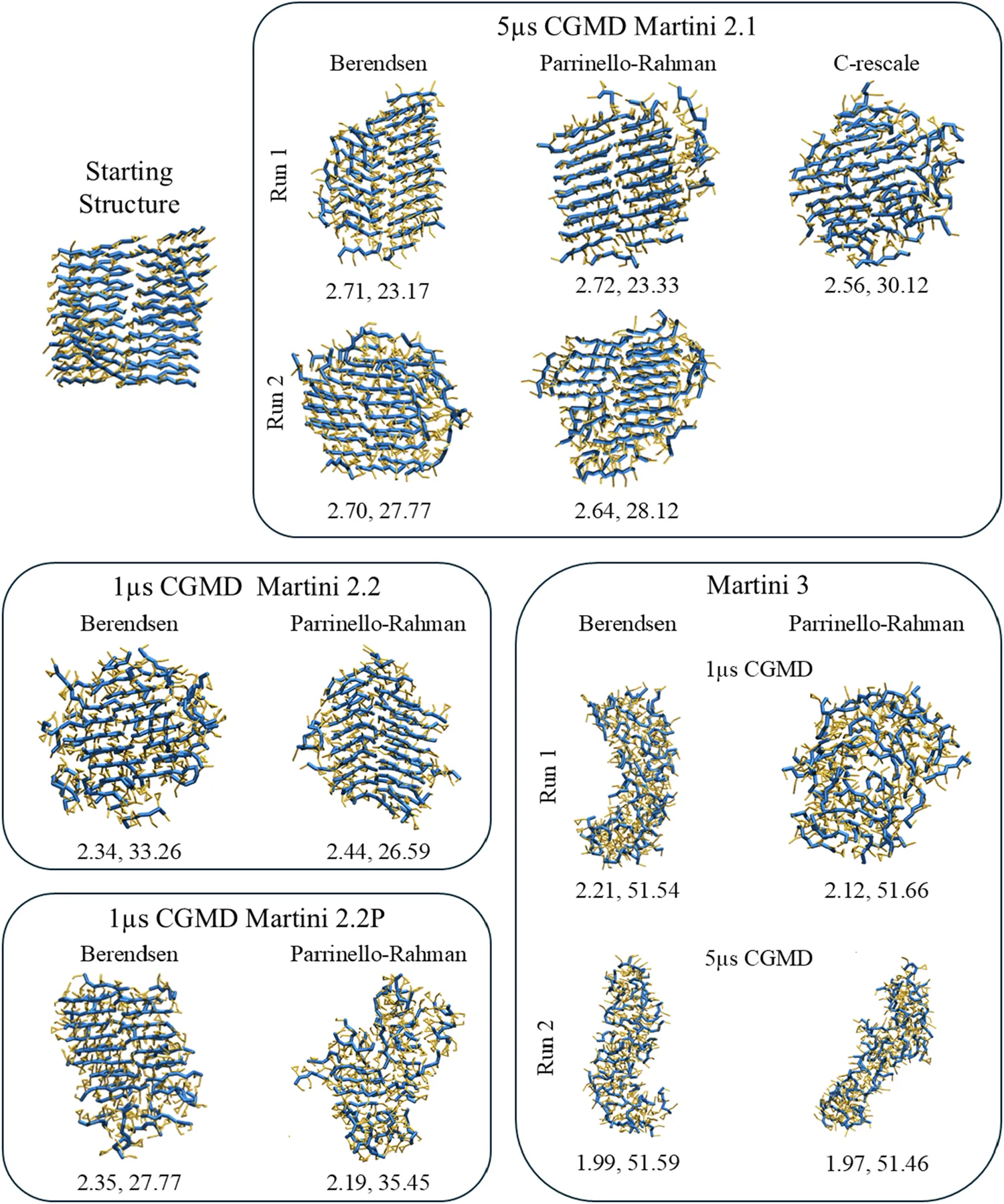
Final frames from the CGMD of the FKFEFKFE self-assembled structure at the end of the simulation durations indicated in the inset. Two simulations were conducted for MARTINI 3. The backbone is colored blue, and the side chains are in yellow. The AP score and configurational entropy per backbone bead in J/mol/K from the last 960 ns of the trajectory are provided below the structure. A higher AP score and a lower entropy support better-ordered structures. A peptide was found to detach from the self-assembled structure in Martini 2.2 (both barostats), Martini 2.2P (both barostats), and Martini 3 with the Parrinello–Rahman barostat, which has been omitted for clarity.

In both Martini 2.1 and 2.2, the outer antiparallel β-sheet layer from the helical structure exhibited more pronounced rearrangement and disorder, while the parallel β-sheet better preserved its self-assembled structure (Figures S9 and S10). Martini 2.2 also maintained the ordered structure, but to a lesser extent compared to 2.1, leading to a drop in AP score to around 2.4 and an increase in entropy per backbone bead to 33.26 and 26.59 J/mol/K using the Berendsen and Parinello–Rahman barostat, respectively. The downward trend in AP score throughout the trajectory shows that the structure may further deviate from the self-assembled structure (Figure 3). Also, the edge peptides were prone to detaching completely from the bilayer and reattaching due to the increased hydrophilicity of the phenylalanine side chains in Martini 2.2, which explains the spikes in RMSD (Figure S11). Interestingly, the Martini 2.2P simulation using the Berendsen barostat is similar to the Martini 2.2 simulation, whereas the Martini 2.2P simulation with the Parrinello–Rahman barostat gradually deformed the self-assembled structure into an amorphous aggregate, increasing the entropy per backbone bead to 35.45 J/mol/K (Figure 2).

**Figure 3:**
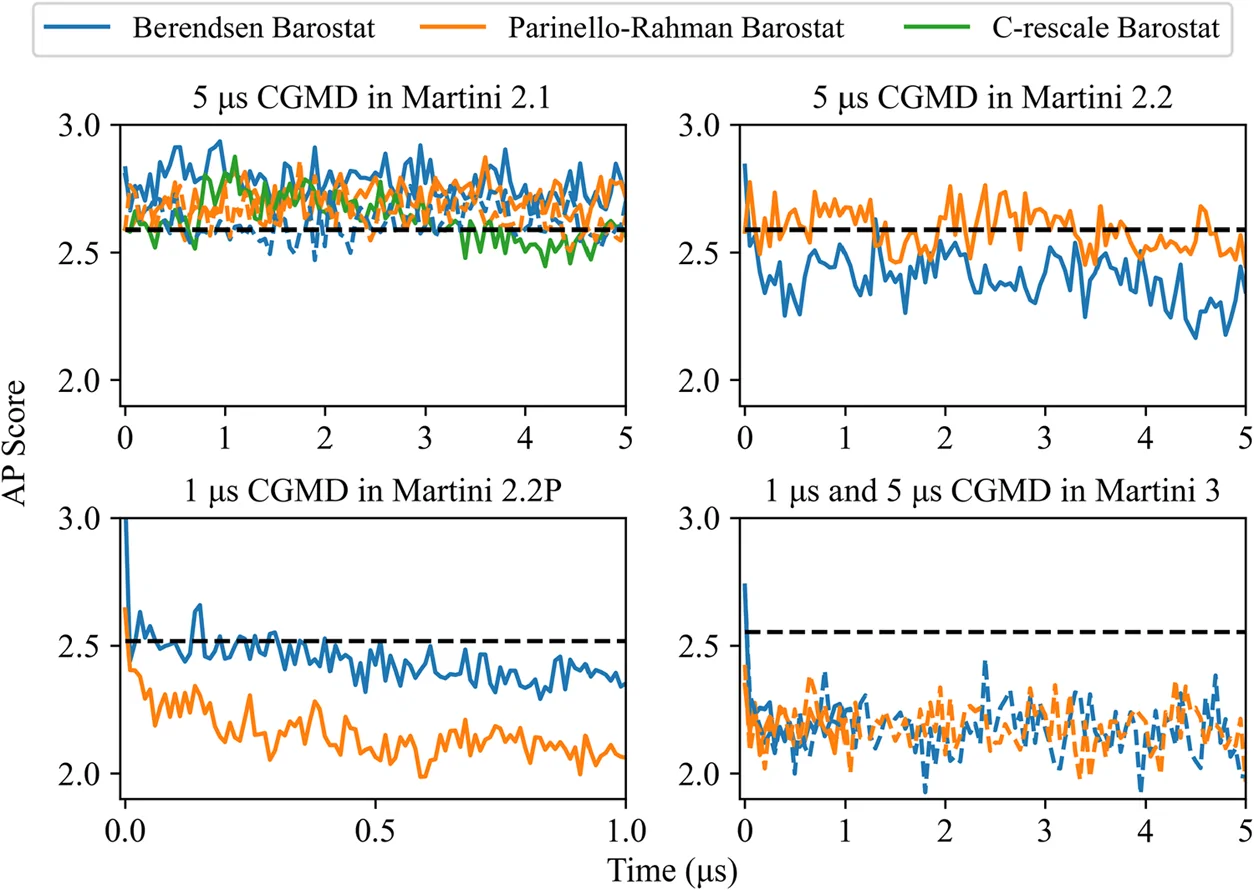
AP score throughout the trajectory, calculated by dividing the SASA of 40 peptides (559.52 nm^2^ for Martini 2 and 553.56 nm^2^ for Martini 3) by the SASA of the system given in Figure S7. The second set of 5 μs simulations with Martini 2.1 and 3 is shown with dashed lines.

In contrast, Martini 3 simulations (regardless of the barostat) transformed the ordered bilayer structure into a clumped aggregate within a few nanoseconds, as seen from the lowest AP score around 2.2 throughout the trajectory (Figure 3). This is ascribed to the elevated hydration of Martini 3, which was also found to depress the aggregation for a variety of peptides compared to Martini 2.2.^61^ The disorder in the Martini 3 CGMD was also captured by the highest entropy per backbone bead of 51.5 J/mol/K, which doubles from that of the Martini 2.1 simulations. In general, the Berendsen barostat performed better in most cases, except for Martini 2.2. The choice of the Martini force field has a far greater influence on the stability of the self-assembled structure in CGMD, with Martini 2.1 consistently preserving the bilayer structure.

### Martini 2.1 Reproduced the Self-Assembly of 20 mM FKFEFKFE Peptides

The 5 μs CGMD of 100 FKFEFKFE peptides (around 20 mM) initially randomly arranged in a 20 nm box (Figure 4A) was performed with Martini 2.1, 2.2, and 3 using the Berendsen barostat. Experimentally, FKFEFKFE is known to self-assemble into nanoribbons at a concentration of 1 mM.^62^ The ∼20 mM concentration used in the simulations is well above the critical aggregation concentration (CAC) of FKFEFKFE. Typically, MD simulations employ artificially high concentrations, 10–100 times the CAC, to accelerate the self-assembly process.^53,61^ Under much higher concentrations, we also observed fast aggregation into disordered structures for the octapeptides. However, their reorganization into ordered assembly was computationally demanding. Therefore, the concentration of ∼20 mM was employed here.

**Figure 4:**
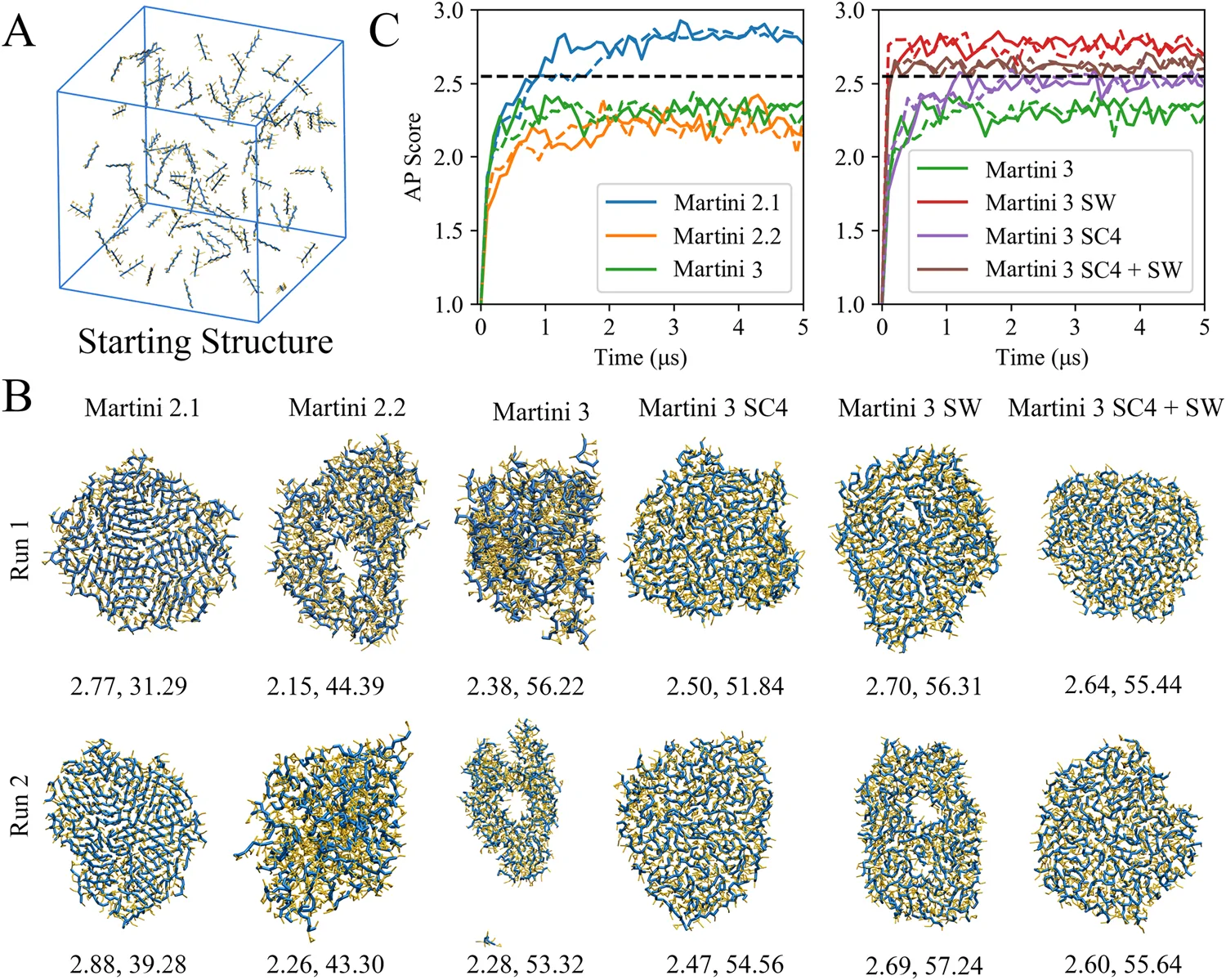
CGMD of 100 FKFEFKFE peptides using the Berendsen barostat. (A) The starting structure with 100 FKFEFKFE peptides randomly arranged in a 20 nm cubic box, and (B) the final frames at the end of 5 μs. Two parallel runs were conducted for each system. The backbone beads are colored blue, and the side chains are yellow. The AP score and configurational entropy per backbone bead in J/mol/K from the last 1000 ns of the trajectory are included. (C) The AP score throughout the trajectory was calculated by dividing the SASA of 100 peptides (1398.8 nm^2^ for Martini 2 and 1383.9 nm^2^ for Martini 3) by the SASA at any simulation time point (Figure S13). In Martini 3 SW, a small water (SW) bead was employed. In Martini 3 SC4, the SC4 bead type (rather than the default TC5 bead) was used for the phenylalanine side chain, along with the regular water bead. In Martini 3 SC4 + SW, both changes were included. The second set of 5 μs simulations with Martini 2.1 and 3 is shown with dashed lines.

Inspired by the reports that restoring the bead type of the phenylalanine side chain beads to those in Martini 2.1 ^27^ and the employment of small water models^30^ are essential for Martini 3 in terms of the self-assembly for the FF dipeptide, we conducted three modifications to the Martini 3 simulations: one using small water, another modifying the bead type of the phenylalanine side chain (2 TC5 and 1 SC4 beads) to those in Martini 2.1 (3 SC4 beads), and last, a combination of the two.

In Martini 2.1, the peptides self-assembled into ordered bilayers with the peptides resembling β-sheets with the phenylalanine side chains arranged in the hydrophobic interior, while the glutamate and lysine residues were exposed to the hydrophilic aqueous environment. In contrast, the peptides formed amorphous aggregates in Martini 2.2 and 3. The ordered structure of the peptide backbones in Martini 2.1 is evident from the pronounced peaks at 0.5, 0.8, and 1 nm in their RDF, indicative of the ordered assembly (Figure S12).^63^ Such peaks are remarkably depressed in Martini 2.2 and 3, ascribed to the increased disorder in the peptide backbones. The small water beads and phenylalanine side chain modifications to Martini 3 lead to a disc-like bilayer structure (with an elevated AP score) as opposed to amorphous aggregates in the unmodified Martini 3. However, the Martini modifications could not achieve an ordered backbone arrangement like Martini 2.1, as evident from the high entropy values (Figure 4B) and the backbone RDF resembling the original Martini 3 simulation (Figure S12). Compared to the phenylalanine side chain modification, the use of small water beads had a greater positive influence on the peptide self-assembly, as evident from a higher AP score.

An AP score greater than 2 clearly indicates peptide aggregation (Figure 4C), which was considered a good indicator for peptide self-assembly.^17^ However, among Martini 2.1, 2.2, and 3, only Martini 2.1 attained SASA below 540 nm^2^ (estimated value for a 100-peptide bilayer based on the 216 nm^2^ SASA for the 40-peptide bilayer). The small water bead simulations in Martini 3 also reached a SASA below 540 nm^2^ (Figure S13). Although the AP scores of 2.77 for Martini 2.1 and 2.70 for Martini 3 SW are similar, Martini 3 SW shows significantly higher configurational entropy per bead: 55 vs 35 J/mol/K for Martini 2.1. We demonstrate the convergence of the configurational entropy per bead by calculating it for growing time windows in Figure S2. Therefore, the configurational entropy can better capture the backbone disorder in longer peptides and is in good agreement with the observed final structures (Figure 4B).

The dynamics of the trajectories revealed that in Martini 2.1, aggregates consisting of a few peptides formed around 10 ns. At around 200 ns, these formed small bilayers, and by 1 μs, all the smaller bilayers merged into a single extended bilayer (Figures S3 and S4). The Martini 2.2 CGMD gradually formed globular aggregates of a few peptides, which then combined into larger aggregates, forming a single aggregate. Such a self-assembly process was found to be much faster for Martini 3: globular aggregates of a few peptides formed over a few nanoseconds, which grew into larger aggregates by 100 ns and merged into one aggregate by 1 μs. The Martini 3 SC4 simulation followed a similar trajectory but formed a disc-like bilayer by 1 μs instead of the disordered aggregate. Martini 3 (SW and SC4 + SW) further accelerated this process, and the disc-like bilayer formed at around 200 and 150 ns, respectively (Figures S5 and S6).

### CGMD of the FKFEFKFE Nanotube

We find that the CGMD of the FKFEFKFE nanotube supports the observations from the CGMD of the FKFEFKFE bilayer. Although the nanotube was not well preserved in Martini 2.1, the bilayers were still maintained, whereas Martini 3 completely deformed the constituent bilayers (Figure 5). Weeks et al. investigated the effects of N-terminal acetylation on FKFEFKFE self-assembly.^62^ They found that both FKFEFKFE and Ac-FKFEFKFE form β-sheet-rich self-assembled structures, with FKFEFKFE forming flat nanoribbons as opposed to helical ribbons formed by Ac-FKFEFKFE. This agrees with the flattening of the curved FKFEFKFE bilayers in Figures 2 and 5.

**Figure 5:**
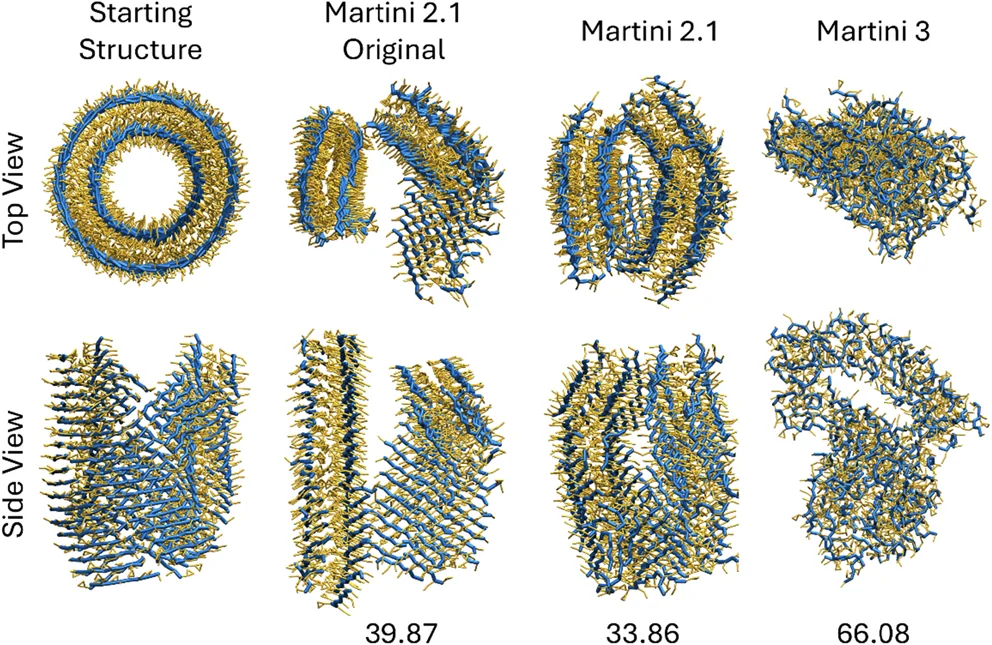
CGMD of the nanotube portion in the PDB structure 7LQG using Martini 2.1 and 3 with the Berendsen barostat. “Martini 2.1 Original” is the CGMD of the Martini 2.1 topology generated by the original *martinize.py* script with the original backbone bond (400 kJ/mol/nm^2^) and angle (25 kJ/mol/rad^2^) force constants, while “Martini 2.1” used 1250 kJ/mol/nm^2^ and 20 kJ/mol/rad^2^ force constants. The configurational entropy per backbone bead in J/mol/K is given below each structure. The backbone is colored blue, and the side chains are colored yellow.

## Discussion

Though amorphous aggregates are readily observed in AAMD and CGMD, self-assembly into ordered structures, such as bilayers, nanotubes, and nanoribbons,^64,65^ is computationally challenging for AAMD starting from a random initial configuration and for CGMD without a proper setup and protocol. Despite exponential growth in high-performance computing, most progress has been in parallelization, driven by advances in multicore processors and graphics processing units. Parallelization enables the simulation of increasingly large systems in MD. However, the number of achievable iterations, *i.e*., the total simulation time, is still bounded by the maximum clock speeds of the processors, since many MD algorithms are serial and long-range interactions in the system limit the possible domain decompositions for parallelization. The reduced degrees of freedom and the use of short-range potentials in CG potentials make the free energy landscape of the systems smoother than in all-atom force fields. In addition to the computational speedup, the smoother landscape allows peptides to traverse more easily, avoiding local minima as seen in AAMD. Even today, most screening studies for self-assembling peptides only look for aggregation in short simulations with durations of 50–400 ns, which are enough to observe aggregation.^18^ However, a long CGMD of about 1 μs simulation time (4 μs effective time) is required to truly observe the formation of self-assembled structures, particularly for longer peptides.

Since the inception of the AP score, it has been acknowledged that it cannot distinguish between self-assembly, crystallization, precipitation, and aggregation, and only measures the initial stages of self-assembly.^17,18^ Still, previous studies focused primarily on screening for aggregation and maximizing the AP score^19^ rather than self-assembly into ordered structures, such as β-sheets and α-helical coiled-coil structures as observed in longer peptides.^66^ Improved descriptors, such as the aggregation detection index, sheet formation index, and tube formation index, have been proposed to quantify self-assembled moieties in CGMD from the RDF.^67^ However, the sheet formation index failed for the CGMD structures obtained here due to the lack of ideal geometry. Therefore, we introduce the use of configurational entropy to characterize peptide self-assembly. A comparison of the configurational entropy supports an approximate threshold of less than 40 J/mol/K for ordered structure. Another feature to qualitatively characterize the ordered structure is the RDF, where patterned peaks exist in the RDF curves for ordered structures.^63^ As indicated in Figures S8 and S12, the periodic peaks exist in the RDF of the peptide backbone beads in Martini 2.1, supporting their ordered arrangement, which becomes weaker in Martini 2.2 and negligible in Martini 3 and its modifications, suggesting the disappearance of the ordered assembly.

The Martini force field was developed using a building-block approach, calibrating the CG bead parameters against oil–water partitioning coefficients.^29^ Thus, Martini is excellent at replicating the hydrophobic effect, especially from bulk solvent effects. There have been multiple reports of Martini 2 overestimating hydrophobic interactions, and thus, Martini 3 was developed to remedy this. Unfortunately, Martini 3 can no longer reproduce short peptide self-assembly, as demonstrated with the FF dipeptide system.^27,30^ From this study, we can assert that the same is true for the amphipathic octapeptide FKFEFKFE and, possibly, for other longer peptides. An important consideration to note is that Martini 3 does not aggregate the FF dipeptide at all. In contrast, in this study, the FKFEFKFE octapeptides aggregate in Martini 3 but fail to exhibit self-assembly into an ordered arrangement. What is more interesting is that the difference between the amino acid particle definitions in Martini 2.1 and 2.2 is the hydrophobicity (SC4 → SC5) of the aromatic side chains, which leads to different hydration energy with the water beads. Still, the minute change has a profound impact on the peptide self-assembly.

Martini 3 redefines all bead types compared to Martini 2 and introduces the small and tiny bead types; thus, a side-by-side comparison with Martini 2 is challenging. Martini 3 offers access to a broader range of monomers, molecules, and solvents due to the definition of smaller beads. For example, Martini 3 represents 2 methanol molecules with a small SP2r bead, whereas Martini 2 does not include a representation of methanol because of its small size. The smaller, differently sized beads in the amino acid definitions of Martini 3 increase the degrees of freedom of the peptides compared to Martini 2. Hence, for long peptides, the folding of individual peptide chains becomes a competing process to the self-assembly, which is not present in the dipeptides and tripeptides. The backbone disorder is absent in shorter FF dipeptide self-assembly and was thus not considered by previous studies, which, nevertheless, becomes important in longer peptides. The Martini 3 simulations of FKFEFKFE here show that the peptide chains collapse and fold on themselves before the interpeptide aggregation starts. The tiny TC5 beads in phenylalanine impose additional restrictions on solvent interactions and introduce artificial free-energy barriers for regular water.^68^ This work indicates that subtle changes in the side chain and solvent parameters impact the reproduction of peptide self-assembly. The issues with Martini 3 may be rectified in later versions of Martini or the polarizable water model for Martini 3. A critical evaluation of the recently released Martini3-IDP^69^ and Go̅Martini 3 ^70^ and future versions for peptide self-assembly is warranted. Enhanced sampling and simulated annealing could also be tackled in future studies.

The experimentally reported nanostructures from β-sheet-forming peptides are highly limited. Another example is the nanotube assembled using Ac-KIIILK-NH_2_ (PDB ID 9CZ3).^71^ Interestingly, the sequence Ac-KIIILK-NH_2_ self-assembles into nanotubes, whereas the reversed sequence Ac-KLIIIK-NH_2_ forms nanosheets (no data deposited in PDB),^71^ which demands more experimental and computational investigation. The CGMD of other peptides, for instance, α-helical peptides, should also be considered for benchmarking when their experimentally solved self-assembled structures become available.

Accurate and reliable Martini simulations using the recommendations from this study should ideally maintain the stability of the self-assembled structure and self-assemble the free peptides into the most favorable structure. The peptides are expected to naturally adopt favorable registry patterns and alternative β-sheet organizations during the course of the simulations, which may be corroborated with experimental results. In cases with an ensemble of favorable self-assembled structures, the CGMD should exhibit indications of multiple clusters with distinct arrangements. Multiple runs with different initial conditions are expected to capture the ensemble of favorable structures. Therefore, researchers working on self-assembling peptides can utilize the recommended simulation protocol (Martini 2.1 with Berendsen barostat) to prospectively screen candidate peptides or predict the relative stability of different self-assembled arrangements by following the protocol described in the Methods section of “CGMD of 100 FKFEFKFE Self-Assembly Process” and “CGMD of FKFEFKFE Bilayer and Nanotube”, respectively. Selecting appropriate termini, side-chain protonation states, and secondary structure from experiments such as circular dichroism and Fourier transform infrared (FTIR) during the simulation setup can significantly improve the accuracy of CGMD and speed of convergence.

## Conclusion

The AAMD revealed that neutral peptide termini via neutral protecting groups or protonation are critical for the stability of self-assembled structures. In FKFEFKFE, the neutral termini are essential for the curvature and widening of the strands from the side-by-side association of the protofilaments in the self-assembled bilayer structure. We hypothesize that the pH of the system is the primary driving force behind the observed variations in diameter and self-assembly in the Cryo-EM structures. That said, the unprotected peptides with charged termini would suppress the self-assembly into large fibrils.

The systematic evaluation of the Martini versions and the barostats shows that the former plays a decisive role in preserving the ordered self-assembled structure, with Martini 2.1 outperforming the other versions, and the Berendsen barostat slightly exceeds the others for Martini 2.1. Martini 2.1 is also successful in simulating the self-assembly process for the octapeptide starting from a random arrangement, which Martini 2.2 and 3 fail to accomplish. The use of small water and modifications to the phenylalanine side chain in the Martini 3 potential improved the final structure from disordered aggregates to bilayer discs, with small water demonstrating greater influence; nevertheless, the backbones could not achieve the ordered arrangement observed in Martini 2.1 and Cryo-EM.

We find that while the AP score is ideal for describing amorphous aggregation, it is inadequate to identify the self-assembly into ordered structures. This can be alleviated by the introduction of configurational entropy. When more experimental structures are available, the generality of the recommended simulation protocols and the implication of configurational entropy in quantifying ordered self-assembly could be further justified. The present findings emphasize the need for standardized and reproducible simulation workflows for peptide self-assembly. Such workflows are important not only for direct mechanistic studies but also for computational screening and emerging AI/ML-based discovery efforts. Improving methodological comparability should therefore help make simulation-derived data sets more reliable for identifying new self-assembling peptide sequences and relating predicted aggregation behavior to ordered supramolecular structure.

## Supplementary Material



## Notes

Supporting figures on the chemical structure of the various FKFEFKFE termini, convergence of configurational entropy, snapshots from the CGMD of the peptide self-assembly process, SASA, RDF, and RMSD throughout the trajectory (PDF)

## Abbreviations

AAMD: all-atom molecular dynamics
AP: aggregation propensity
C-rescale: stochastic cell rescaling
CG: coarse-grained
CGMD: coarse-grained molecular dynamics
MD: molecular dynamics
PDB: protein data bank
RDF: radial distribution function
RMSD: root-mean-square deviation
SASA: solvent accessible surface area
V-rescale: velocity rescaling
