# Supplementary material for: Amphipathic β‑Sheet-Forming Octapeptide Self-Assembly Using the Martini Potential Family
Source: ACS Omega. 2026 Aug 12;11(33):49330–40. doi: 10.1021/acsomega.6c02457 (PMC13520551; doi:10.1021/acsomega.6c02457)
Supplement: Supplementary file 1 [file ao6c02457_si_001.pdf]

Supporting Information

Amphipathic β-sheet-forming Octapeptide Self-Assembly Using the Martini Potential Family

Dibyajyoti Maity* and Baofu Qiao*

Department of Natural Sciences, Baruch College, City University of New York,

New York, New York 10010, United States

Email: baofu.qiao@baruch.cuny.edu

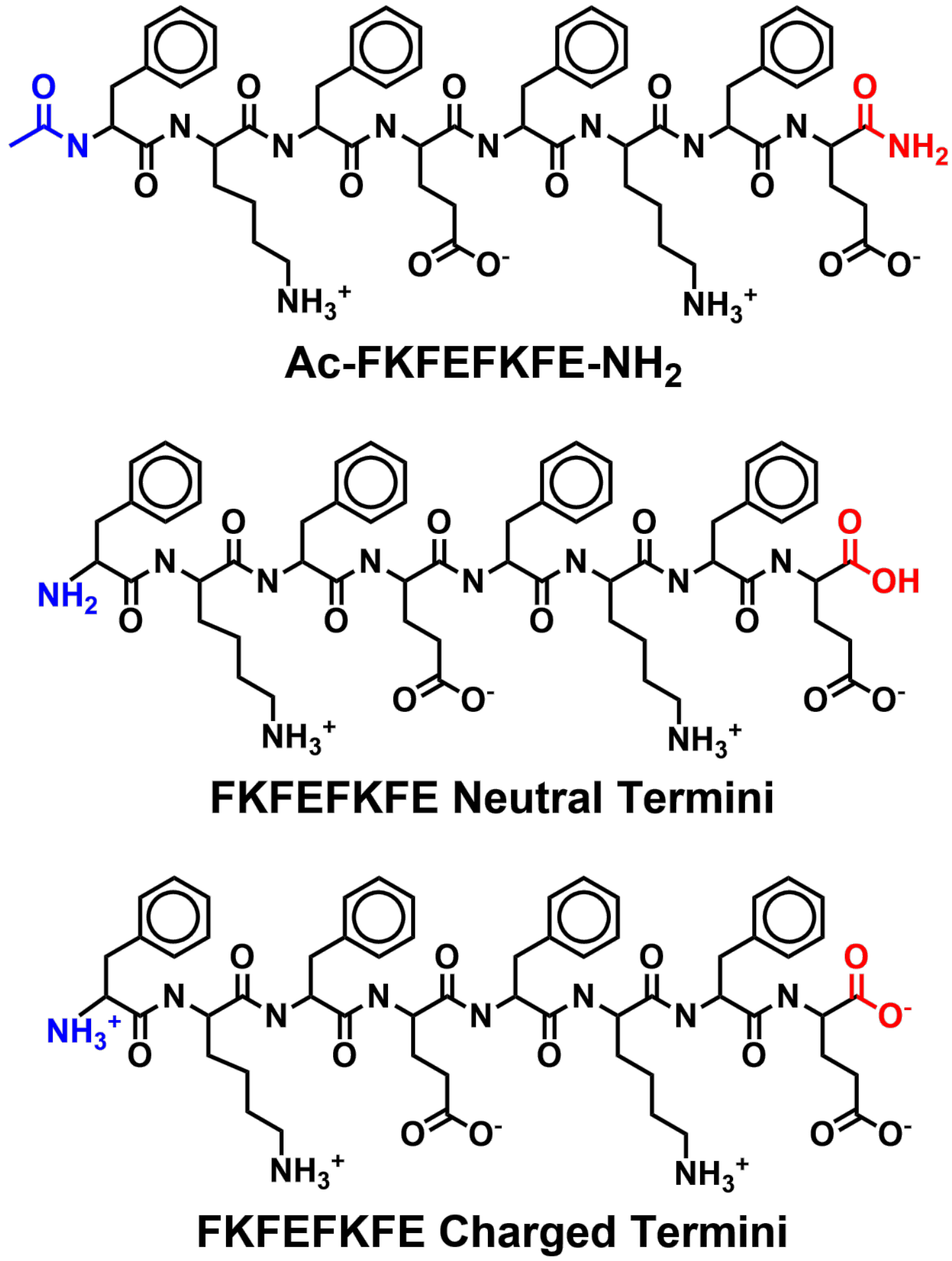

Figure S1. Chemical structure of the FKFEFKFE peptide with the three different termini: protected (N-acetyl and C-amide), neutral (NH_2 and COOH), and charged termini (NH_3^+ and COO^-). The N- and C-termini are colored in blue and red, respectively.

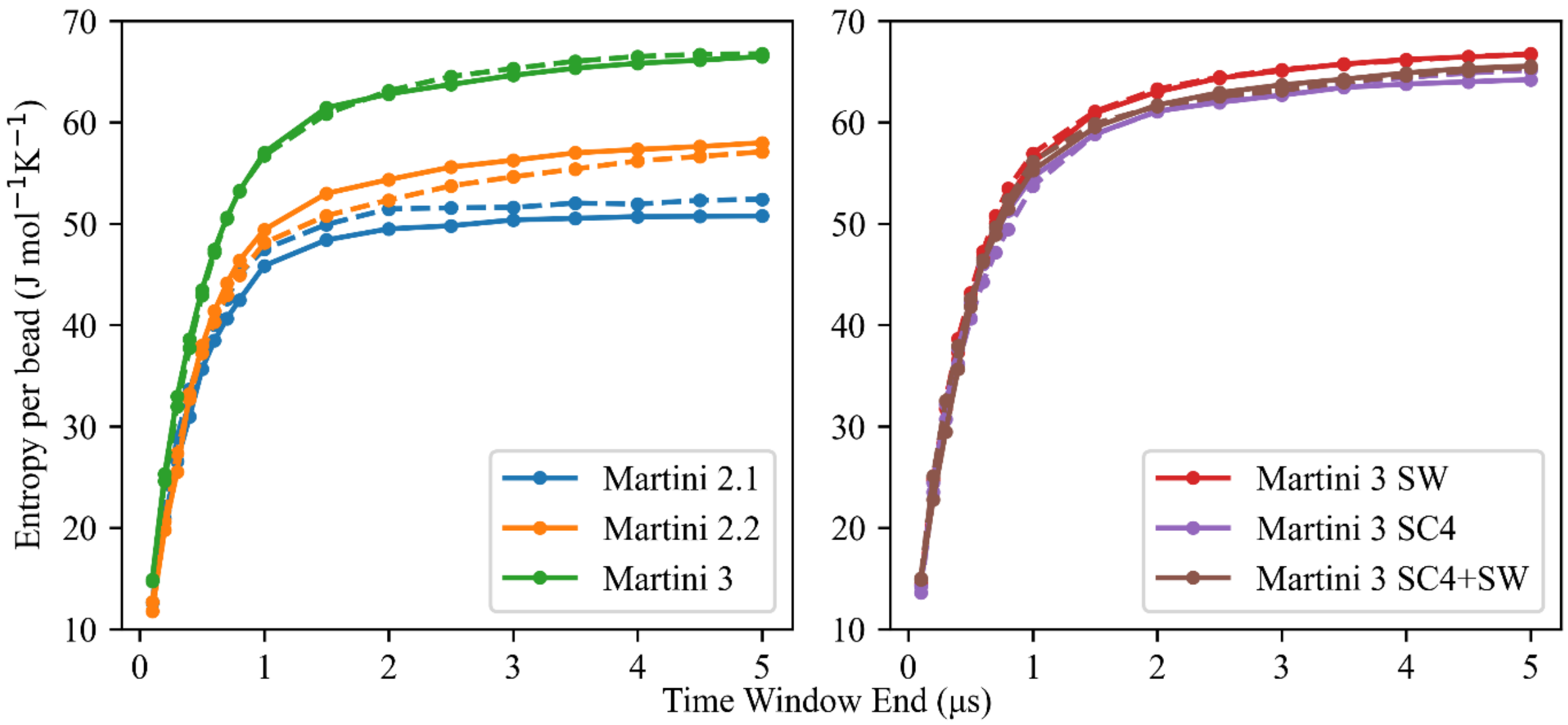

Figure S2: Convergence of configurational entropy per bead from the CGMD of 100 FKFEFKFE peptides in a 20 nm cubic box. The entropy was calculated using growing time windows, starting at 0 μs and ending at the times corresponding to the x-coordinates of the data points. The second set of 5 μs simulations is shown with dashed lines. Note that the final entropy in the plots above is higher than in Figure 4 because it is calculated for the entire 5 μs trajectory rather than the last 1000 ns.

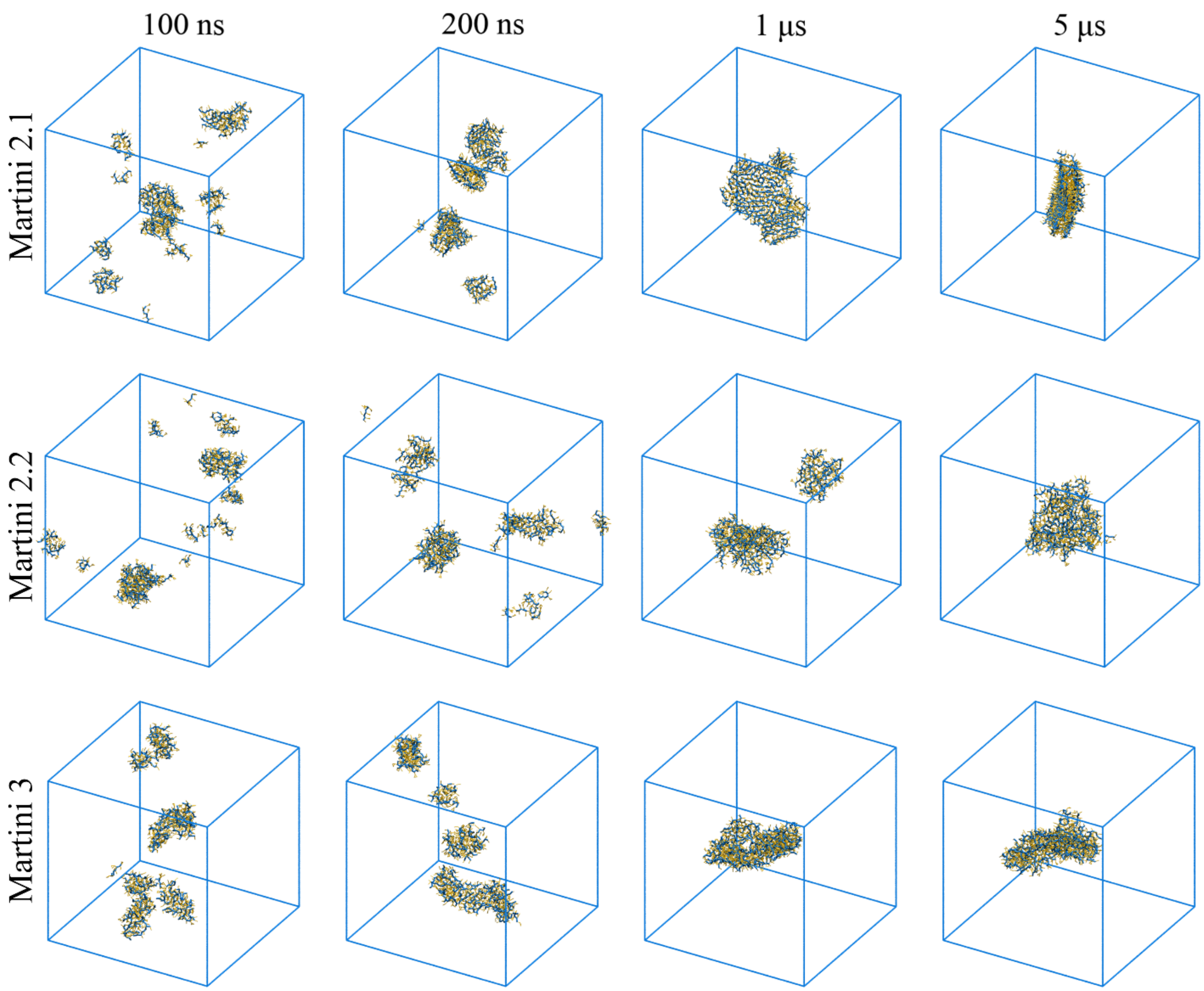

Figure S3. Self-assembly process of FKFEFKFE simulated using Martini 2.1, 2.2, and 3. Snapshots from the CGMD of 100 peptides in a 20 nm cubic box at 100 ns, 200 ns, 1 μs, and 5 μs. The backbone is colored blue, and the side chains are yellow.

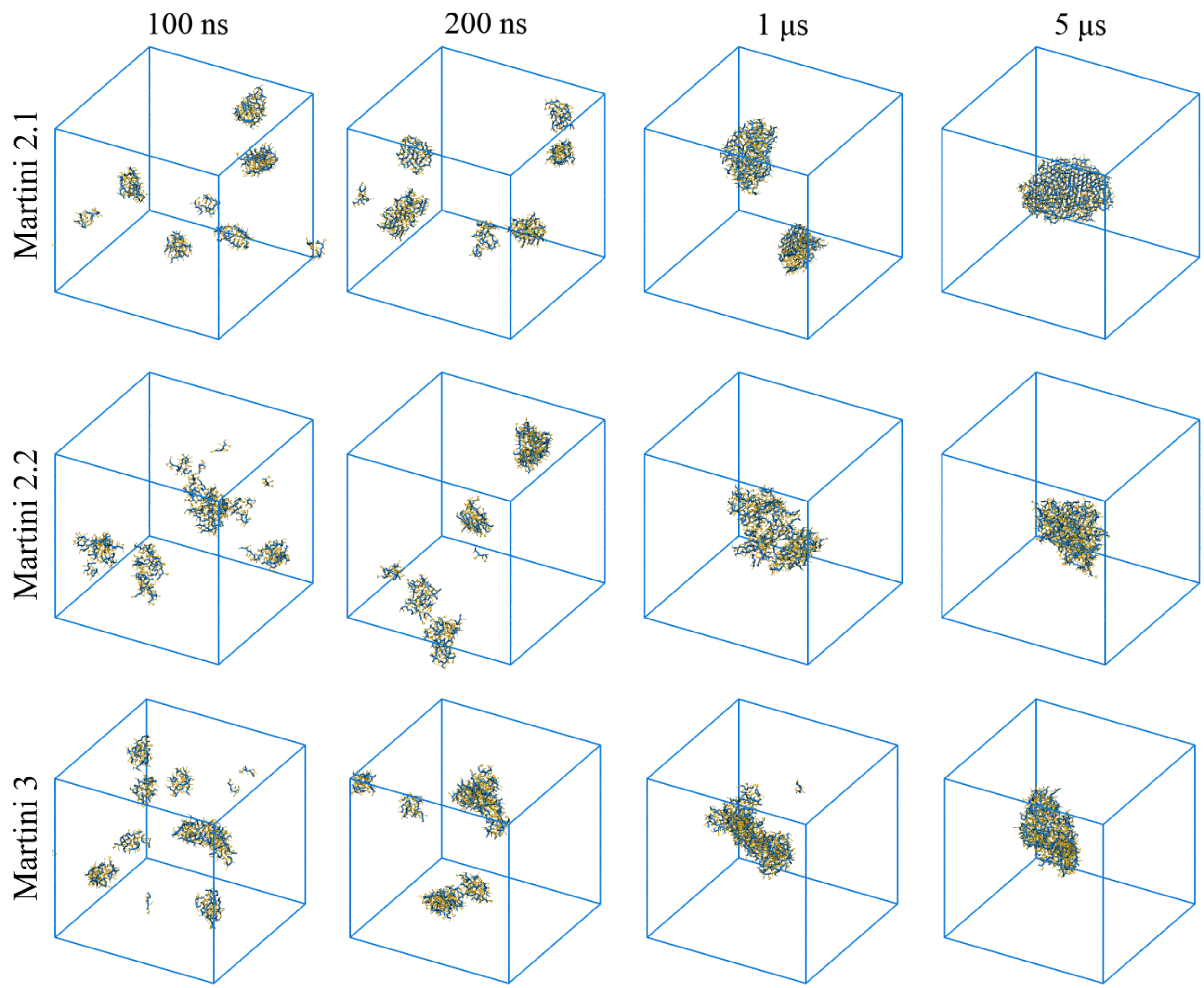

Figure S4. Same as Figure S3, but for the second set (parallel run) of CGMD simulations.

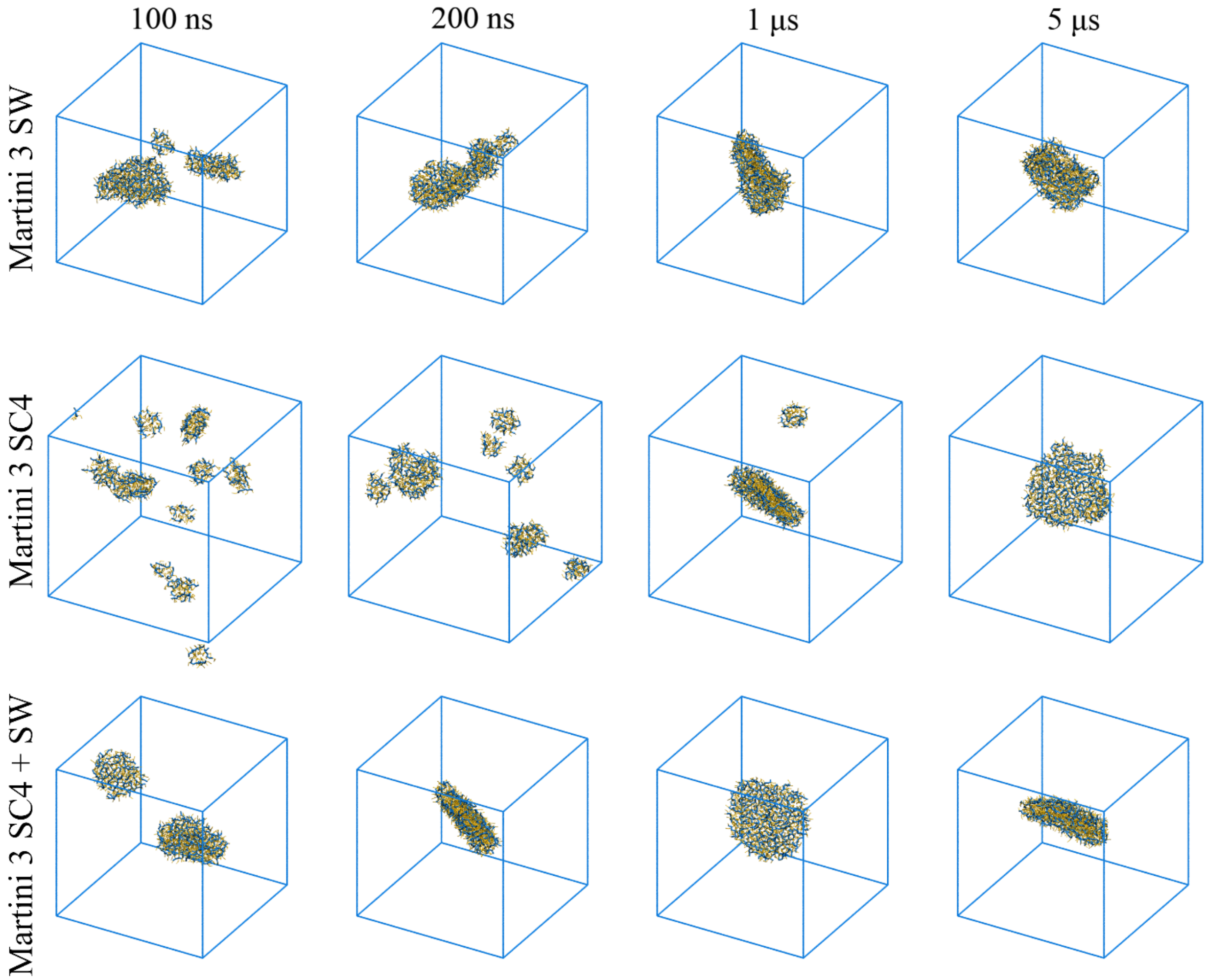

Figure S5. Self-assembly process of FKFEFKFE simulated using Martini 3 with modifications. Snapshots from the CGMD of 100 peptides in a 20 nm cubic box at 100 ns, 200 ns, 1 μs, and 5 μs. The backbone is colored blue, and the side chains are yellow. Martini 3 SW is the simulation with a small water bead (SW) instead of regular water (W). Martini 3 SC4 is the simulation in regular water where the TC5 beads in the phenylalanine side chain are modified to SC4. Martini 3 SC4 + SW is the simulation combining the two changes.

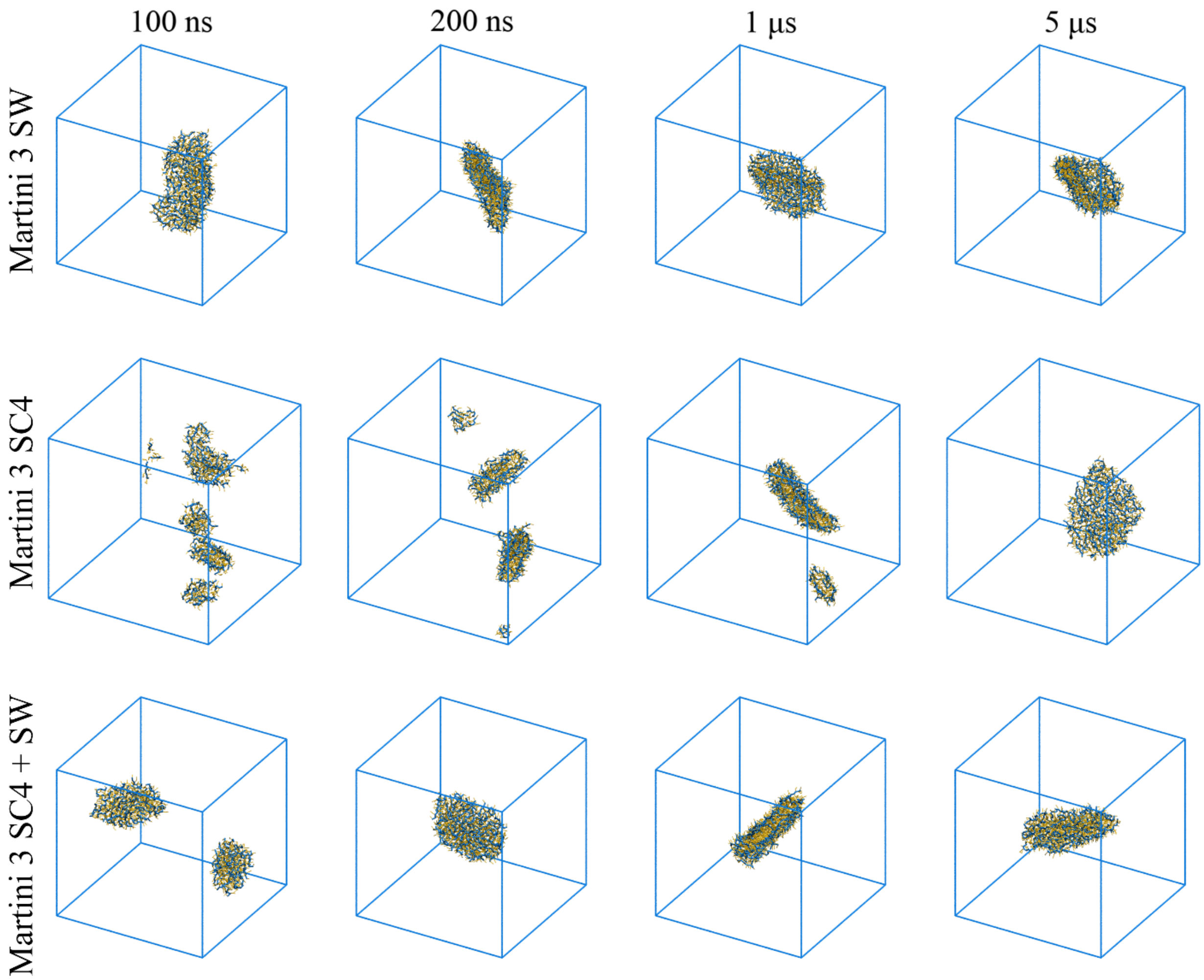

Figure S6. Same as Figure S5, but for the second set (parallel run) of CGMD simulations.

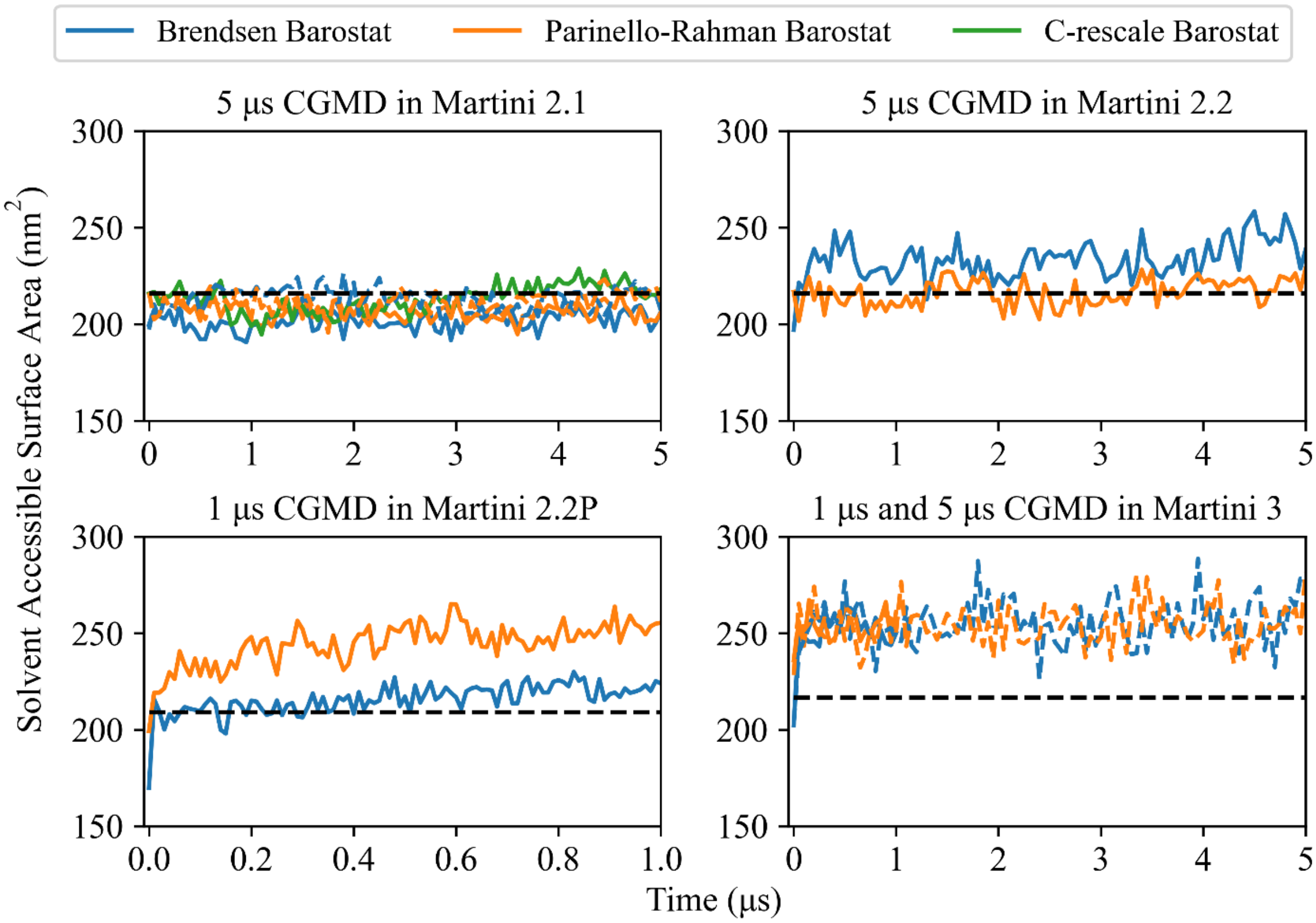

Figure S7. SASA from the CGMD of the FKFEFKFE self-assembled structure. The dashed line at 216 nm^2 is the SASA of the reference structure. All three Martini 2.1 simulations, Martini 2.2 with Parinello-Rahman barostat, and Martini 2.2P with Berendsen barostat maintained a SASA close to or below that of the reference structure. In comparison, disorder and deformation from the starting structure were observed in the other simulations with a higher SASA. The second set of 5 μs simulations with Martini 2.1 and 3 are shown with dashed lines.

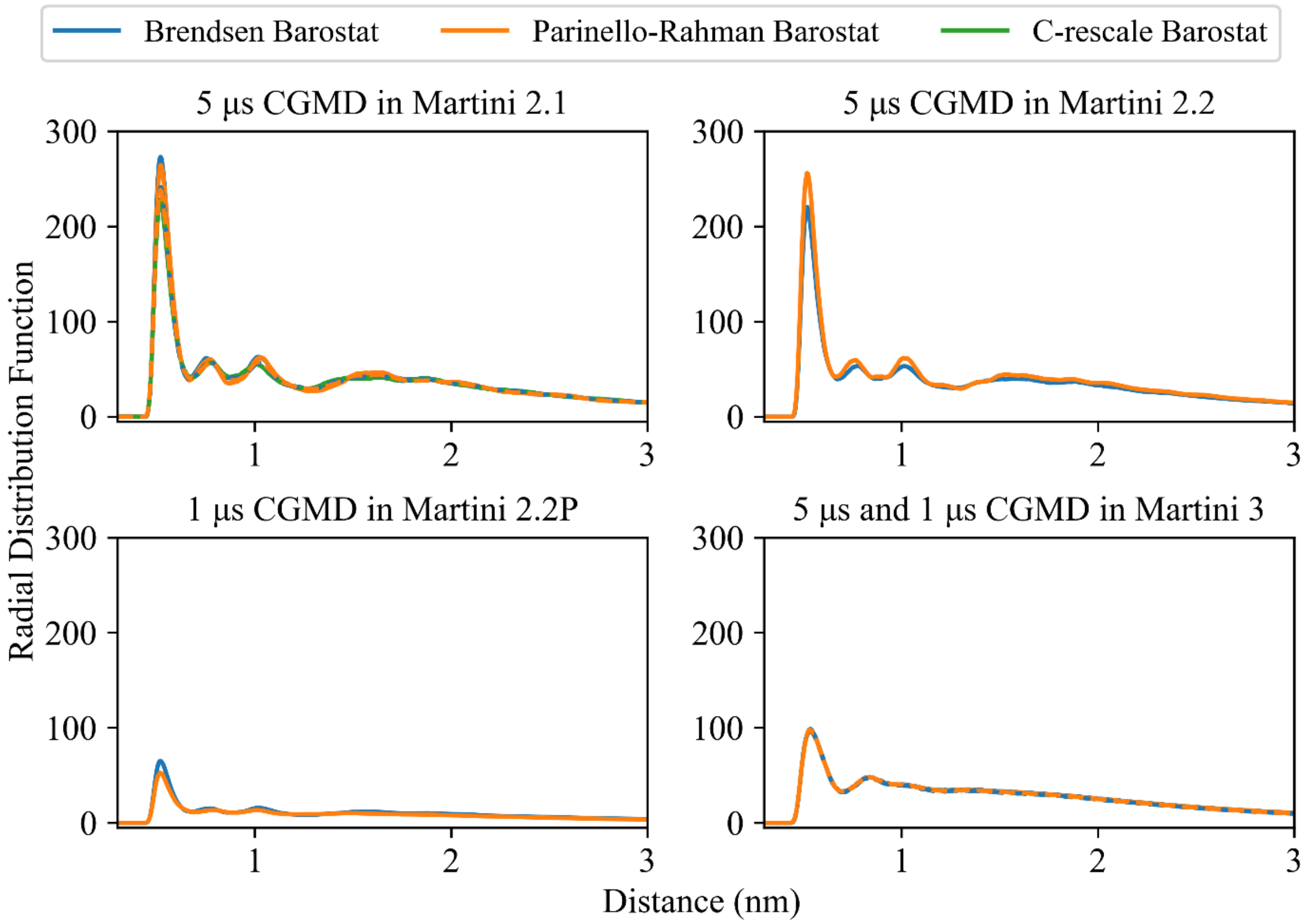

Figure S8. The radial distribution function of the backbone beads from the CGMD of the FKFEFKFE self-assembled structure. The second set of 5 μs simulations with Martini 2.1 and 3 are shown with dashed lines.

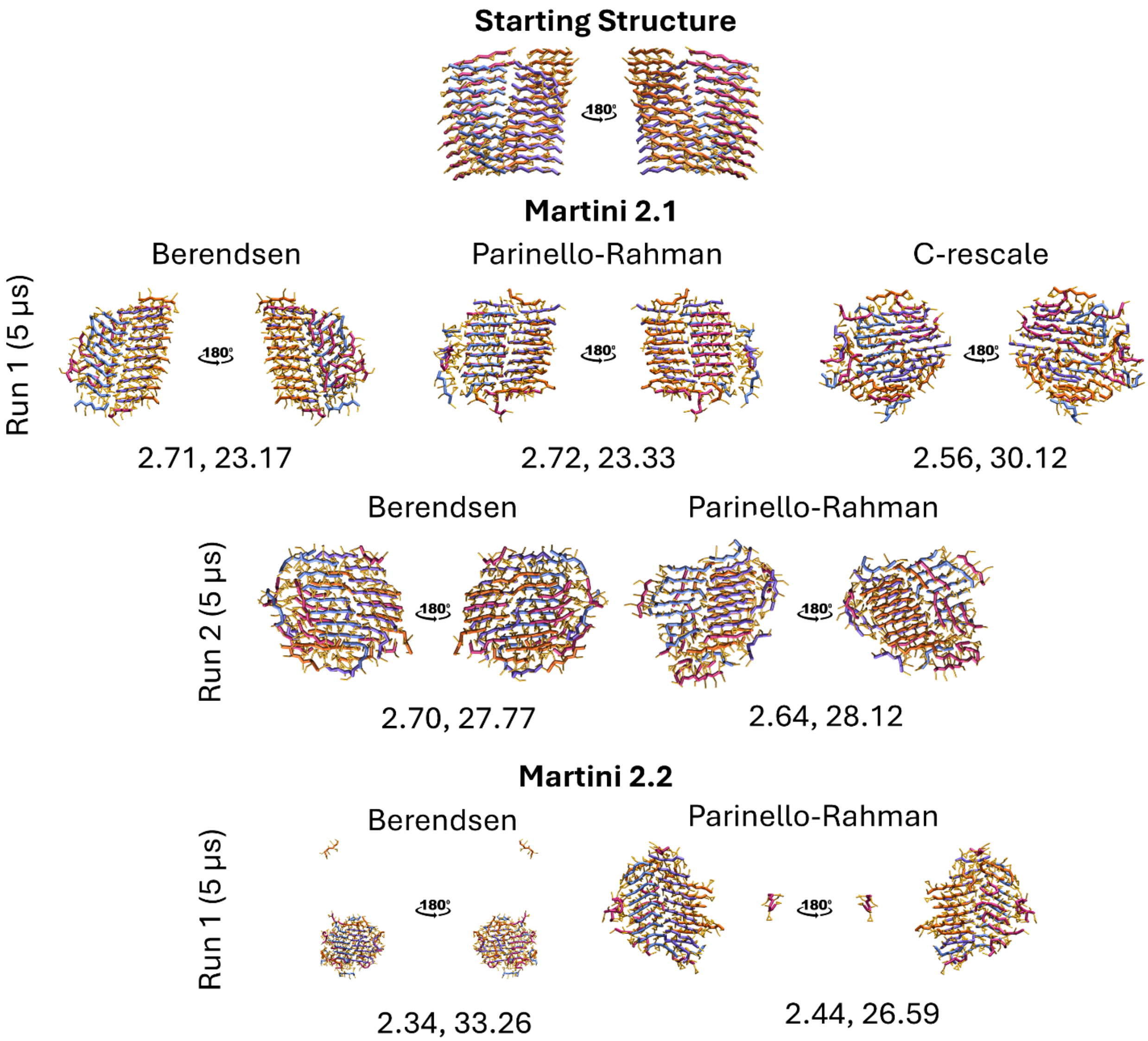

Figure S9. Final frames from the CGMD of the FKFEFKFE self-assembled structure at the end of 5 μs for Martini 2.1 and 2.2. The backbone of the two inner β-sheets is colored blue and purple, while the two outer β-sheets are colored orange and pink. The side chains are yellow. The AP score and the configurational entropy per bead of the backbone in $J \cdot mol^{-1}K^{-1}$ from the last 960 ns of the trajectory are provided below the structure. A higher AP score and a lower entropy support a better-ordered structure.

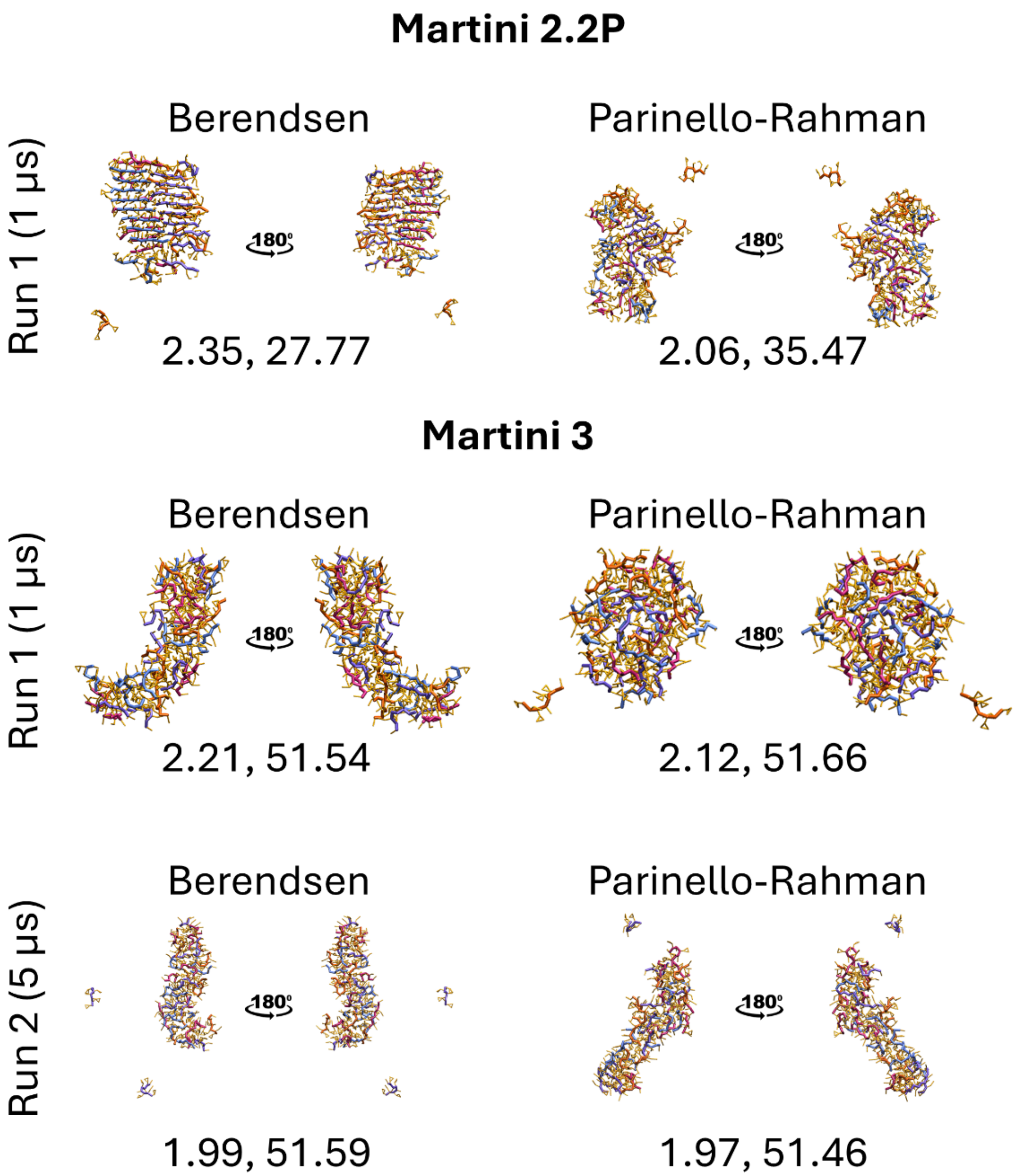

Figure S10. Similar to Figure S9, but for Martini 2.2P and 3.

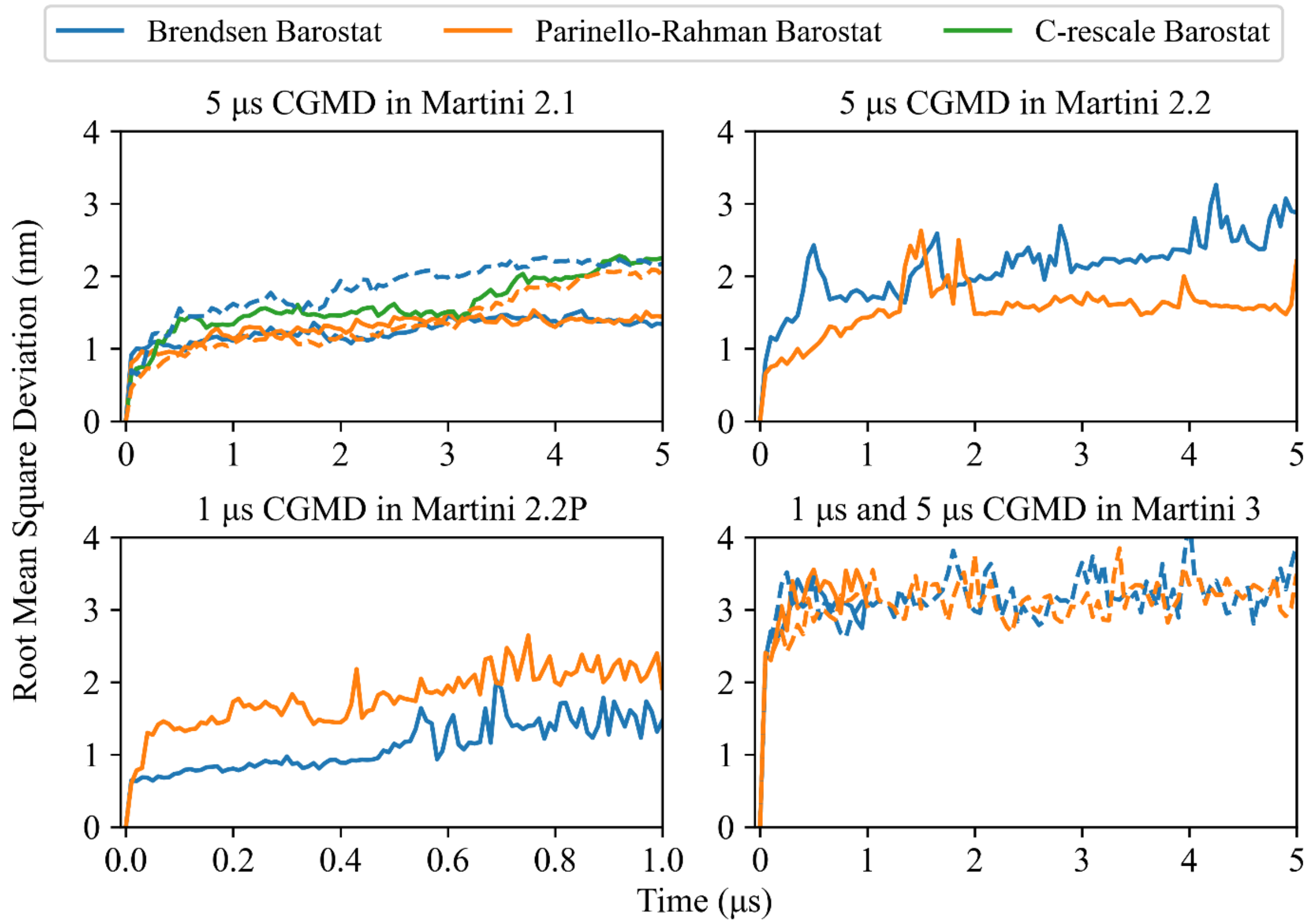

Figure S11. Root mean square deviation (RMSD) from the CGMD of the FKFEFKFE self-assembled structure. The second set of 5 μs simulations is shown with dashed lines.

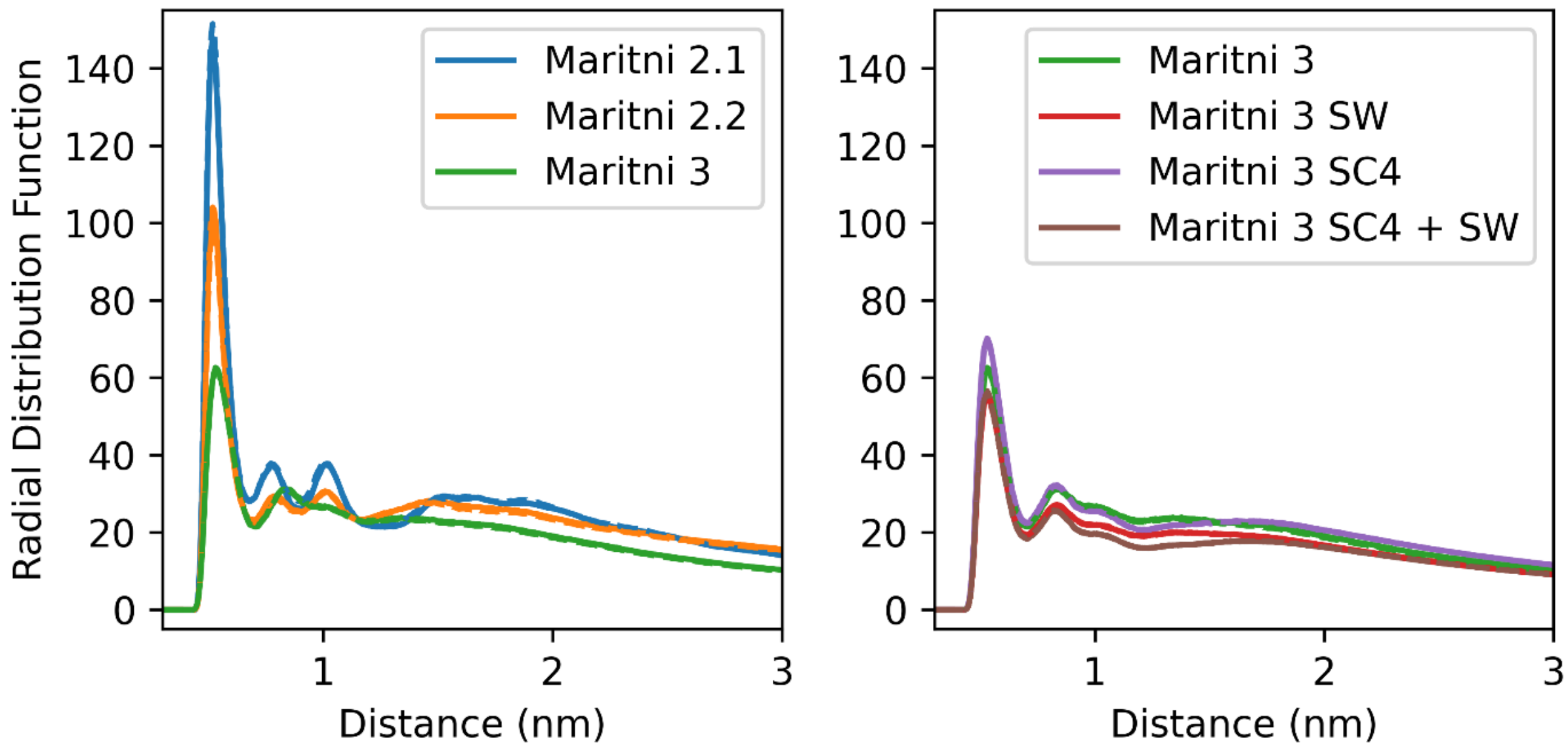

Figure S12. The radial distribution function of the backbone beads from the CGMD of 100 FKFEFKFE peptides in a 20 nm box. The second set of 5 μs simulations is shown with dashed lines.

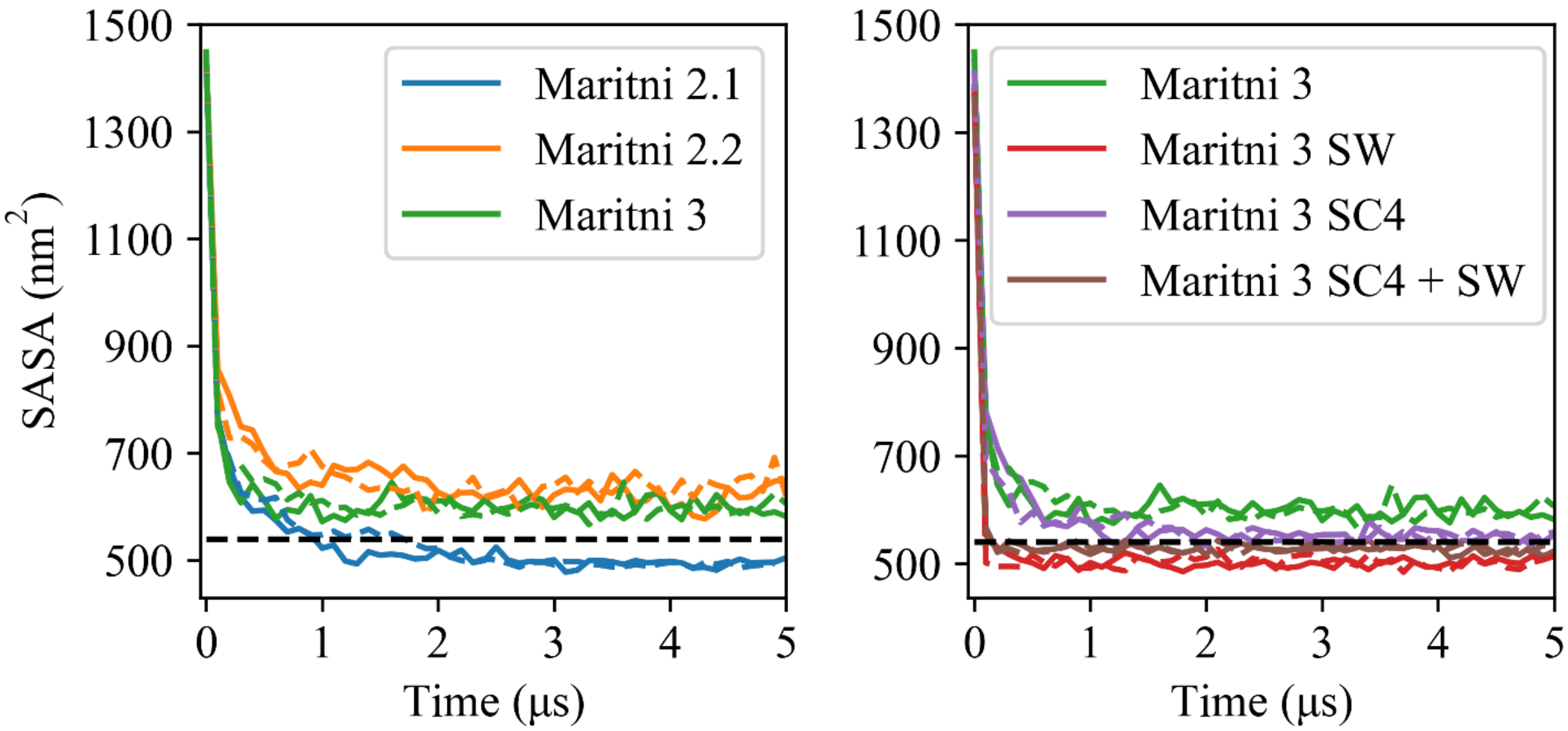

Figure S13. SASA from the CGMD of 100 FKFEFKFE peptides in a 20 nm box. The dashed line at 540 nm^2 is the SASA estimated for the self-assembled structure of 100 peptides, given that the FKFEFKFE self-assembled structure from the Cryo-EM structure containing 40 peptides has a SASA of 216 nm^2. The second set of 5 μs simulations is shown with dashed lines.
